# Impairment of the mitochondrial one-carbon metabolism enzyme SHMT2 causes a novel brain and heart developmental syndrome

**DOI:** 10.1007/s00401-020-02223-w

**Published:** 2020-10-05

**Authors:** Àngels García-Cazorla, Edgard Verdura, Natalia Juliá-Palacios, Eric N. Anderson, Leire Goicoechea, Laura Planas-Serra, Enkhtuul Tsogtbaatar, Nikita R. Dsouza, Agatha Schlüter, Roser Urreizti, Jessica M. Tarnowski, Ralitza H. Gavrilova, SHMT2 Working Group, Alfonso Oyarzábal, Inés Medina, Aida Ormazábal, Jordi Muchart, Juan Manuel Carretero, Cristina Jou, Mireia del Toro, Andrés Nascimento, Abraham J. Paredes, Delia Yubero, Roser Colomé, Montserrat Ruiz, Agustí Rodríguez-Palmero, Stéphane Fourcade, Benjamin Cogné, Thomas Besnard, Marie Vincent, Stéphane Bézieau, Clifford D. Folmes, Michael T. Zimmermann, Eric W. Klee, Udai Bhan Pandey, Rafael Artuch, Margot A. Cousin, Aurora Pujol

**Affiliations:** 1 Neurometabolic Unit and Synaptic Metabolism Lab, Neurology Department, Institut Pediàtric de Recerca, Hospital Sant Joan de Déu, and MetabERN, 08950 Barcelona, Catalonia Spain; 2 Centre for Biomedical Research on Rare Diseases (CIBERER), Instituto de Salud Carlos III, 28029 Madrid, Spain; 3 Neurometabolic Diseases Laboratory, Bellvitge Biomedical Research Institute (IDIBELL), L’Hospitalet de Llobregat, 08908 Barcelona, Catalonia Spain; 4 Department of Pediatrics, Children’s Hospital of Pittsburgh, University of Pittsburgh School of Medicine, Pittsburgh, PA 15224 USA; 5 Department of Cardiovascular Medicine, Mayo Clinic, Scottdale, AZ 85260 USA; 6 Department of Biochemistry and Molecular Biology, Mayo Clinic, Scottdale, AZ 85260 USA; 7 Department of Molecular Pharmacology and Experimental Therapeutics, Mayo Clinic, Scottdale, AZ 85260 USA; 8 Bioinformatics Research and Development Laboratory, Genomic Sciences and Precision Medicine Center, Medical College of Wisconsin, Milwaukee, WI 53226 USA; 9 Clinical Biochemistry Department, Institut de Recerca Sant Joan de Déu, and MetabERN, 08950 Barcelona, Catalonia Spain; 10 Department of Clinical Genomics, Mayo Clinic, Rochester, MN 55905 USA; 11 Department of Neurology, Mayo Clinic, Rochester, MN 55905 USA; 12 Department of Pediatrics, Paediatric Neurology Unit, University Hospital Germans Trias i Pujol, 08916 Badalona, Catalonia Spain; 13 Service de Génétique Médicale, CHU de Nantes, and INSERM, CNRS, Université de Nantes, l’Institut du Thorax, Nantes, 44000 Nantes, Pays de la Loire France; 14 Clinical and Translational Science Institute, Medical College of Wisconsin, Milwaukee, WI 53226 USA; 15 Center for Individualized Medicine, Mayo Clinic, Rochester, MN 55905 USA; 16 Department of Health Sciences Research, Mayo Clinic, Rochester, MN 55905 USA; 17 Catalan Institution of Research and Advanced Studies (ICREA), 08010 Barcelona, Catalonia Spain

**Keywords:** SHMT2, Mitochondrial one-carbon metabolism, Congenital microcephaly, Perisylvian polymicrogyria, Cardiomyopathy

Inborn errors of metabolism cause a wide spectrum of neurodevelopmental and neurodegenerative conditions [15]. A pivotal enzyme located at the intersection of the amino acid and folic acid metabolic pathways is SHMT2, the mitochondrial form of serine hydroxymethyltransferase. SHMT2 performs the first step in a series of reactions that provide one-carbon units covalently bound to folate species in mitochondria: it transfers one-carbon units from serine to tetrahydrofolate (THF), generating glycine and 5,10-methylene-THF [4, 11, 12].

Using whole exome sequencing (WES), we identified biallelic *SHMT2* variants in five individuals from four different families. All identified variants were located in conserved residues, either absent or extremely rare in control databases (gnomAD, ExAC), and cosegregated based on a recessive mode of inheritance (pRec = 0.9918 for this gene) (Supplementary Figs. 1–3, Supplementary Table 1). In family F1, a homozygous missense variant present in two affected siblings was located in a region without heterozygosity (~ 10 Mb, the only region > 1 Mb shared by both siblings) in which no other candidate variants were found, providing a strong genetic evidence of causality for these variants. The missense/in-frame deletion nature of these variants, and the absence of loss-of-function homozygous individuals in control databases, combined with the fact that complete loss of SHMT2 is embryonic lethal in the mouse [18], suggested that these variants may cause hypomorphic effects. Using 3D molecular dynamics models of the SHMT2 protein, we concluded that these candidate variants probably alter the SHMT2 oligomerization process, and/or disrupt the conformation of the active site, thus inducing deleterious effects on SHMT2 enzymatic function (Supplementary Figs. 4–8, Supplementary Tables 2–3, Supplementary video) [8, 19].

All patients presented a similar phenotype, characterized by dysmorphic features including long palpebral fissures, eversion of lateral third of lower eyelids, arched eyebrows, long eyelashes, thin upper lip, long philtrum, short fifth finger, fleshy pads at the tips of the fingers, mild 2–3 toe syndactyly and low-set thumbs. All patients exhibited intellectual disability and motor dysfunction, in the form of spastic paraparesis, ataxia, and/or peripheral neuropathy. Also, four out of five patients showed hypertrophic cardiomyopathy or atrial-septal defects, which tend to progress over time. All of the patients showed congenital microcephaly; MRI revealed corpus callosum abnormalities in all patients and perisylvian polymicrogyria-like pattern in patients P1–P4 (Fig. 1a–c, Supplementary Figs. 9–11, Supplementary Table 4). Quadriceps muscle and myocardium biopsies from Patient 4 showed myopathic changes, and myocardium biopsy showed the presence of “ragged red” fibers, suggestive of defective mitochondria (Fig. 1d, Supplementary Fig. 12).

**Fig. 1:**
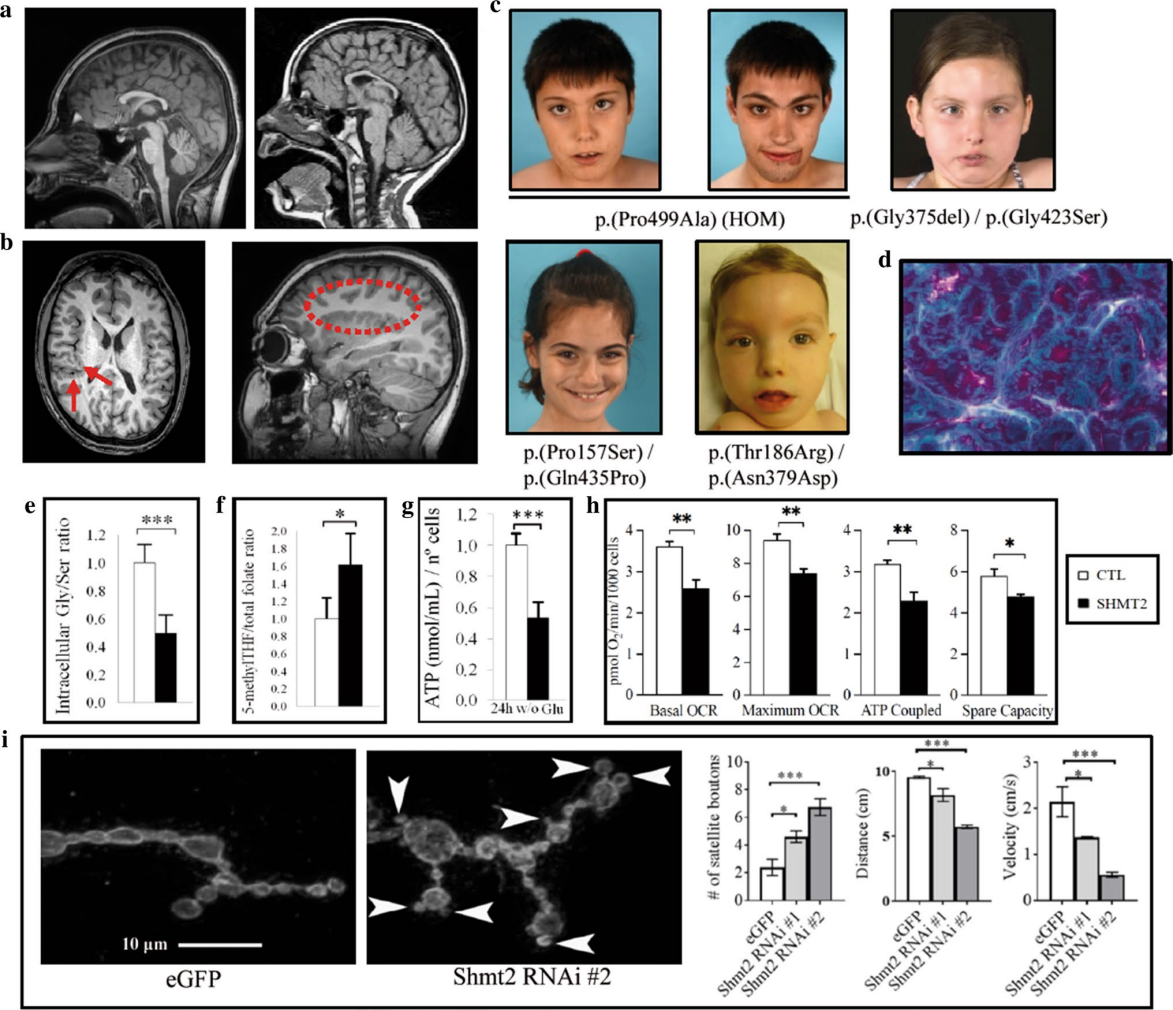
SHMT2-mutated patients: phenotype and functional evaluation in fibroblasts. **a** Sagital T1-weighted MRI planes showing corpus callosum hypoplasia in Patient P1 (left) and Patient P4 (right). **b** Axial T1-weighted MRI planes (left) in Patient P2 showing perisylvian polymicrogyria (PMG) visible around both Sylvian fissures and insulae (arrows) and right parasagittal T1-weighted MRI (dotted contour). Note the stippled gray-white boundary of the polymicrogyric cortex compared to the smooth gray-white boundary in normal cortical areas. **c** Dysmorphic features in Patients P1–P5. **d** Modified Gomori trichrome staining of Patient 4 myocardium biopsy sample showed the presence of “ragged red” fibers, consistent with a mitochondrial cytopathy. **e** Quantification of Gly/Ser ratio in fibroblasts from control individuals (CTL, *n* = 6) and patients (SHMT2, *n* = 5). **f** 5′-Methyl THF (tetrahydrofolate) normalized to total folate levels in fibroblasts from control individuals (CTL, *n* = 5) and patients (SHMT2, *n* = 5). **g** Measure of ATP concentration in control individuals (CTL, *n* = 6) and patients (SHMT2, *n* = 5) fibroblasts after 24-h incubation in a medium without glucose. Values were normalized by number of cells. Quantification depicted as fold change to control fibroblasts. **h** Quantification of mitochondrial oxygen consumption rates (OCR, pmol O_2_/min/1000 cells) in control individuals (CTL, *n* = 5) and patients (SHMT2, *n* = 4). **i** *Shmt2* knockdown in motoneurons cause neuromuscular junction (NMJ) and motility defects in Drosophila. Left: immunofluorescence images of the neuromuscular junctions of muscle 4 segment A2–A3 stained with the presynaptic marker horseradish peroxidase (HRP, arrowhead). Right: quantification of the average number of satellite boutons, climbing distance and velocity in control (eGFP) and mutant (Shmt2 RNAi #1, #2) flies (*n* = 15-18 for boutons, *n* = 30 for distance and velocity). In **e**–**h**, values are expressed as mean ± SD, and two-tailed Student *t* tests were performed; in **i**, values are expressed as mean ± SEM, and one-way ANOVA with Tukey’s multiple comparisons test was performed (*p < 0.05, **p < 0.01, ***p < 0.001)

To assess the pathogenicity of SHMT2 variants, we pursued functional testing with patient-derived primary fibroblasts. SHMT2 protein levels in fibroblasts were not significantly altered (Supplementary Fig. 13). While all metabolites were in the normal range in plasma, fibroblasts from affected individuals showed a significant decrease in glycine/serine ratios compared to controls. Folate metabolism was also impaired: 5-methyltetrahydrofolate levels were increased in patients in relation to total folate (Fig. 1e, f). The substrate of SHMT2 tetrahydrofolate (THF) was undetectable in mitochondria-enriched control fibroblast samples, but low levels of this molecule were detectable in extracts from patient fibroblasts (Supplementary Table 5). These data support the impairment of SHMT2 enzymatic function in these patients. Because folate and serine are required for proper mitochondrial translation [11, 12], we verified levels of mitochondrial OXPHOS complexes, which did not vary (Supplemental Fig. 14).

Next we analyzed bioenergetic and mitochondrial function in patients’ fibroblasts, which were described to be impaired in knockout human cancer cell lines [11, 12]. ATP measurements, as well as extracellular flux analysis in a Seahorse device, under glucose restriction conditions, indicated an impaired oxidative capacity in patients’ cells relative to controls (Fig. 1g, h, Supplementary Fig. 15). Mitochondrial membrane potential was found to be altered, as well as ROS levels (both total and mitochondrial), supporting mitochondrial redox metabolism malfunction (Supplementary Fig. 16).

In previous works, Shmt2 knockout mice exhibited embryonic lethality, attributed to severe mitochondrial respiration defects in fetal liver, and ensuing inhibition of erythroblast differentiation resulting in anemia. Moreover, metabolic defects were not observed in brain tissue, possibly due to the preferential use of the glycine cleavage system to provide one-carbon units [17]. To investigate whether the patients’ neurological phenotype could be mediated by non-neuronal autonomous mechanisms, we knocked down *Shmt2* specifically in *Drosophila* motor neurons (~ 65% knockdown of Shmt2 RNA as shown previously by qPCR) [3]. We analyzed the morphology of presynaptic terminals at neuromuscular junctions (NMJs), which reliably model excitatory synapses in the mammalian brain and spinal cord [2]. While no changes in the numbers of total boutons or mature boutons were observed in Shmt2-knockdown animals compared with eGFP controls, we found a significant increase in the numbers of satellite boutons, emerging from the main nerve terminal or budding excessively from primary boutons and forming clusters (Fig. 1i, Supplementary Fig. 17). Of note, previous studies have shown increased satellite boutons in *Drosophila* models for Amyotrophic Lateral Sclerosis and Spastic Paraplegia [7, 16]. Moreover, mutant flies showed reduced climbing distance and velocity compared to eGFP control animals (Fig. 1i). These results may reflect a role for human SHMT2 in the maintenance of presynaptic vesicles, and can be attributed to a selective decrease of Shmt2 in neurons, without any significant systemic interfering effects. Thus, these studies argue against non-cell autonomous mechanisms from the periphery causing neuronal malfunction in patients.

Interestingly, this novel rare disease entity corresponds faithfully to an intersection of diverse clinical manifestations associated with defects in metabolic pathways in which SHMT2 plays a crucial role, such as amino acid and folate metabolism and mitochondrial homeostasis [4, 11, 12]. SHMT2 impairment alters intracellular glycine/serine levels, which provides the main source of mitochondrial one-carbon units in proliferating cells, and thus probably contributes to microcephaly and polymicrogyria [9, 10, 13]. Microcephaly associated with hypomyelination is also seen in patients with loss of PYCR2, an enzyme of proline synthesis which interacts with SHMT2, causing hyperglycinemia, underscoring the impact of dysregulated glycine/serine levels on neurodevelopment [6]. SHMT2 malfunction also depletes a downstream product species, 5,10-methylTHF, required for nucleotide metabolism [1, 5]. Microcephaly, developmental delay/intellectual disability and cardiomyopathy have been extensively described in defects of folate metabolism [14]. In summary, we describe a novel neurodevelopmental, syndromic encephalopathy and movement disorder associated with cardiac defects. Despite a certain degree of variable severity, clinical manifestations were consistent in all individuals and thus establish a well-defined and recognizable clinical syndrome of defective folate and amino acid metabolism.

## Electronic supplementary material

Below is the link to the electronic supplementary material.

Supplementary material 1 (MP4 28187 kb)

Supplementary material 2 (PDF 2804 kb)

## References

[1] Acuna-Hidalgo R, Schanze D, Kariminejad A, Nordgren A, Kariminejad MH, Conner P (2014). Neu-Laxova syndrome is a heterogeneous metabolic disorder caused by defects in enzymes of the l-serine biosynthesis pathway. Am J Hum Genet.

[2] Budnik V (1996). Synapse maturation and structural plasticity at Drosophila neuromuscular junctions. Curr Opin Neurobiol.

[3] Celardo I, Lehmann S, Costa AC, Loh SH, Martins LM (2017). DATF4 regulation of mitochondrial folate-mediated one-carbon metabolism is neuroprotective. Cell Death Differ.

[4] Ducker GS, Rabinowitz JD (2017). One-carbon metabolism in health and disease. Cell Metab.

[5] El-Hattab AW (2016). Serine biosynthesis and transport defects. Mol Genet Metab.

[6] Escande-Beillard N, Loh A, Saleem SN, Kanata K, Hashimoto Y, Altunoglu U (2020). Loss of PYCR2 causes neurodegeneration by increasing cerebral glycine levels via SHMT2. Neuron.

[7] Estes PS, Boehringer A, Zwick R, Tang JE, Grigsby B, Zarnescu DC (2011). Wild-type and A315T mutant TDP-43 exert differential neurotoxicity in a Drosophila model of ALS. Hum Mol Genet.

[8] Giardina G, Brunotti P, Fiascarelli A, Cicalini A, Costa MG, Buckle AM (2015). How pyridoxal 5’-phosphate differentially regulates human cytosolic and mitochondrial serine hydroxymethyltransferase oligomeric state. FEBS J.

[9] De Koning TJ, Duran M, Dorland L, Gooskens R, Van Schaftingen E, Jaeken J (1998). Beneficial effects of l-serine and glycine in the management of seizures in 3-phosphoglycerate dehydrogenase deficiency. Ann Neurol.

[10] De Koning TJ, Klomp LWJ (2004). Serine-deficiency syndromes. Curr Opin Neurol.

[11] Minton DR, Nam M, McLaughlin DJ, Shin J, Bayraktar EC, Alvarez SW (2018). Serine Catabolism by SHMT2 Is Required for Proper Mitochondrial Translation Initiation and Maintenance of Formylmethionyl-tRNAs. Mol Cell.

[12] Morscher RJ, Ducker GS, Li SH, Mayer JA, Gitai Z, Sperl W, Rabinowitz JD (2018). Mitochondrial translation requires folate-dependent tRNA methylation. Nature.

[13] Murtas G, Marcone GL, Sacchi S, Pollegioni L (2020). l-serine synthesis via the phosphorylated pathway in humans. Cell Mol Life Sci.

[14] Pope S, Artuch R, Heales S, Rahman S (2019). Cerebral folate deficiency: analytical tests and differential diagnosis. J Inherit Metab Dis.

[15] Saudubray J-M, Baumgartner M, Walter J (2016). Inborn metabolic diseases, diagnosis and treatment.

[16] Sherwood NT, Sun Q, Xue M, Zhang B, Zinn K (2004). Drosophila spastin regulates synaptic microtubule networks and is required for normal motor function. PLoS Biol.

[17] Tani H, Mito T, Velagapudi V, Ishikawa K, Umehara M, Nakada K (2019). Disruption of the mouse Shmt2 gene confers embryonic anaemia via foetal liver-specific metabolomic disorders. Sci Rep.

[18] Tani H, Ohnishi S, Shitara H, Mito T, Yamaguchi M, Yonekawa H (2018). Mice deficient in the Shmt2 gene have mitochondrial respiration defects and are embryonic lethal. Sci Rep.

[19] Walden M, Tian L, Ross RL, Sykora UM, Byrne DP, Hesketh EL (2019). Metabolic control of BRISC–SHMT2 assembly regulates immune signalling. Nature.

