# Supplementary material for: Impairment of the mitochondrial one-carbon metabolism enzyme SHMT2 causes a novel brain and heart developmental syndrome
Source: Acta Neuropathol. 2020 Oct 5;140(6):971–5. doi: 10.1007/s00401-020-02223-w (PMC7665968; doi:10.1007/s00401-020-02223-w)
Supplement: Supplementary file 2 — Supplementary material 2 (PDF 2804 kb) [file 401_2020_2223_MOESM2_ESM.pdf]

SUPPLEMENTAL MATERIAL

METHODS

Family recruitment and sampling

Patients and relatives are recruited and examined at the Neurology Department of Hospital Sant Joan de Déu (Barcelona, Spain) for Families 1 and 3, the Department of Clinical Genomics at Mayo Clinic (Rochester, USA) for Family 2, and the Genetics Department of Centre Hospitalier Universitaire de Nantes (France) for Family 4. Detailed neurological and neurocognitive examination, neurophysiological (brainstem auditory evoked potentials, nerve conduction studies, and electromyography), cardiologic (echocardiography) and ophthalmological studies were conducted. X-ray, brain MRI (T1, T2, and FLAIR) and spectrometric brain imaging were performed on all available patients. Blood samples and skin-derived fibroblast cell lines were obtained using standard methods. Written informed consent was obtained from all participants in this study according to the Declaration of Helsinki.

Study approval

The research project was approved by the Clinical Research Ethics Committee for Research Ethics of IDIBELL (PR076/14), Hospital Sant Joan de Déu Ethics Committee (Project PI17/00109), the Institutional Review Board of Mayo Clinic (IRB# 12-009346) and the CPP Ouest V Committee (Ref MESR DC 2017 2987) for Nantes University Hospital.

Biochemical studies

Plasma, urine, cerebrospinal fluid (CSF), fibroblasts and mitochondrial amino acids were analyzed by UPLC coupled to tandem mass spectrometry, as previously reported [6]. Briefly, amino acids were separated in a Waters ACQUITY UPLC H-class chromatograph and quantified with a Waters Xevo TQD triple-quadrupole mass spectrometer using positive electrospray ionization conditions in MRM mode. 5-Methyltetrahydrofolate was quantified by HPLC with fluorescence detection as previously reported [24], and total folate was quantified by automated chemoimmunoluminiscence (Abbot automated analyzed). All fibroblast samples were prepared following the same protocol. One million cells were homogenized and suspended in 200 µl of PBS, prior to freezing at -80ºC until analysis. After thawing, the supernatant was centrifuged (1500 x g, 10 minutes), collected and analyzed. Supplementary Table 5 shows the amino acid (in µmol/L) and folate species (in nmol/L) concentrations in the cell supernatants.

Whole exome sequencing analysis and variant assessment

Genomic DNA was extracted from blood. Family F1 and F3’s whole-exome sequencing (WES) was performed using the SureSelectXT Human All Exon V5 50 Mb kit (Agilent) for DNA capture and sequencing on a HiSeq 2000 Platform (Illumina) at CNAG (Centre Nacional d’Anàlisi Genòmica, Barcelona). Patient 3 (Family F2) was already reported with clinical WES testing (XomeDx by GeneDx, Gaithersburg, Maryland) on the trio, although a variant in a different gene (*SPOCK1*) was reported [8]. Since this time, however, the ExAC/gnomAD database constraint metrics [17, 19] have shown that *SPOCK1* is not intolerant to variation, and there have been no additional reports of individuals with *de novo SPOCK1* variants or emerging evidence of a Mendelian disease association with this gene. Additionally, deficient mice show no obvious developmental phenotypes [26]. Variants from families F1 and F3 were filtered using the URD-Cat platform (https://rdcat.cnag.crg.eu/). We prioritized nonsynonymous coding variants with a frequency lower than 0.01 in the ExAC, 1000 Genomes and gnomAD databases. Missense variants were evaluated using several predictors (PolyPhen-2, SIFT, and Mutation Taster). Candidate variants were validated and tested for cosegregation in all available family members by Sanger sequencing.

ExAC: http://exac.broadinstitute.org/[20]
gnomAD: https://gnomad.broadinstitute.org/[17]
1000 genomes: https://www.internationalgenome.org/1000-genomes-browsers/[2]
Polyphen-2: http://genetics.bwh.harvard.edu/pph2/[25]
SIFT: https://sift.bii.a-star.edu.sg/[29]
MutationTaster(v2): http://www.mutationtaster.org/[27]

Protein Modeling

Sequence alignment was performed using ClustalOmega (https://www.ebi.ac.uk/tools/msa/clustalo/), with sequences extracted from NCBI databases. We used sequence homology assessed by HMMER3 software [10] to identify SHMT2 structures. The 3D experimental model of human SHMT2 bound to glycine and folate-competitive pyrazolopyran inhibitor bound to the active site (5V7I) was downloaded from RCSB PDB [3, 9, 12]. SWISS-MODEL [4] homology modeling was performed to fill in the unresolved residues. Structures were visualized with PyMOL version 2.2.3 (https//www.pymol.org) to assess the position of the variants in the 3D models of the SHMT2 dimer, and to analyze spatial patterns among structure-based scores. BioR [18] was used to annotate genomic variants, followed by custom scripts to map them onto the protein structure. We assessed the folding energy ($\Delta\Delta G_{fold}$) of SHMT2 using a protein structure-based algorithm, FoldX [28]. Changes in the potential energy of multi-body contacts, which accounts for shape and solvation in a more nuanced way, were assessed [11], as were changes to residue-level frustration (SRLF) [15].

Molecular Dynamics Simulations

Similar to previous works [13, 30, 31], we carried out Molecular Dynamics (MD) using the Generalized Born implicit solvent molecular dynamics (isMD) simulations, which were in NAMD and the CHARMM36 [14] force field. The homology model was utilized for our initial conformation for the WT. *In silico* mutagenesis was performed using FoldX for the mutations and gnomAD variants. We utilized an interaction cutoff of 12Å with strength tapering (switching) beginning at 10Å, a simulation time step of 1fs, conformations recorded every 2ps. Each initial conformation was used to generate 3 replicates, and each replica was energy-minimized for 10000 steps, followed by heating to 300K over 300ps via a Langevin thermostat. A further 12ns of simulation trajectory was generated and the final 10ns were analyzed. All trajectories were first aligned to the initial wild type conformation using Cα atoms. Root mean-squared deviations (RMSD) and root mean-squared fluctuations (RMSF) were calculated using Cα atoms. Principal Component (PC) analysis was performed in Cartesian space. Analysis was carried out using custom scripts, leveraging VMD and the Bio3D R package. We used the approach of Karamzadeh et al [16] to calculate Free Energy Landscapes (FELs) from PC sampling. Protein structure visualization was performed with PyMol and VMD.

Fibroblast cultures

Fibroblast cell lines from the skin biopsy samples of Patients 1-5 and controls were cultured at 37ºC and 5% CO_2 in Dulbecco's modified Eagle's Medium (DMEM) supplemented with 10% fetal bovine serum (FBS) and 100 U/ml penicillin and 100 µl/ml streptomycin. Fibroblast passages ranged from to 10 to 15 in all experiments, and controls were age-matched individuals.

Mitochondrial enrichment

Fibroblast pellets were homogenized in 4 volumes of ice-cold homogenization buffer (HB) with a 1 mL needle at 4ºC. The HB was prepared immediately before use, and it was composed of 225 mM D-mannitol, 25 mM HEPES-KOH, 1 mM EGTA, and protease inhibitors, pH 7.4. The remaining homogenate was centrifuged at 600 x g for 10 min at 4 ºC to precipitate the nuclei and unbroken cells. The supernatant was centrifuged again under the same conditions to

eliminate all debris, and was then centrifuged at 10000 x g for 20 min at 4ºC. The supernatants were enriched in ER, cytosol, lysosomes, and depleted of mitochondria, and were kept separated. The pellets comprised the crude mitochondria or enriched mitochondria fraction.

RNA extraction and quantitative real-time PCR

RNA was extracted from fibroblasts using an RNeasy Mini kit (QIAGEN) and cDNA was synthesized using a Superscript IV kit (Life Technologies) following the manufacturers' instructions. RT-PCR primers to sequence cDNA in Patient 3 were: SHMT2_F: GAGGACCGAATCAACTTTGC and SHMT2_R: CTCACGGAACTGTCGAGAAG

Western Blot

For the Western blot analyses, fibroblasts were homogenized in RIPA buffer (150 mM NaCl, 1% Nonidet P40, 0.5% sodium deoxycholate, 0.1% SDS, and 50 mM Tris, pH 8.0), sonicated for 2 min at 4ºC, centrifuged, mixed with 4x NuPAGE LDS Sample Buffer (Invitrogen) and heated at 70ºC for 10 min. 25-50 μg of protein samples were subjected to polyacrylamide gel electrophoresis at 120 V in NuPAGE MOPS SDS Running Buffer (Invitrogen) supplemented with 5 mM sodium bisulfite (Ref. 243973, Sigma-Aldrich). The proteins were transferred to nitrocellulose membranes using an iBlot 2 Gel Transfer Device (Invitrogen). After blocking in 5% bovine serum albumin (BSA, Sigma-Aldrich), and 0.05% TBS-Tween (TBS-T) for 1 h at room temperature, membranes were incubated with primary antibodies overnight at 4°C. Primary antibodies included: Anti-SHMT2, rabbit (1/1000, ref: HPA020549, Sigma-Aldrich), Anti-NDUFA9, mouse (1/1000, ref:A21344, Invitrogen), Anti-NDUFB8, mouse (1/1000, ref:459210, Invitrogen), Anti-Complex II, mouse (1/1000, ref:A21345, Invitrogen), Anti-Complex III, mouse (1/1000, ref:A21362, Invitrogen), Anti-MTCOI (Complex IV), mouse (1/500, ref:ab14705, Abcam), Anti-Complex IV subunit IV, mouse (1/1000, ref: A21348, Invitrogen), Anti-Complex V, mouse (1/500, ref: A21350, Invitrogen), Anti-VDAC1, rabbit (1/1000, ref: ab15895, Abcam), Anti-β-Actin, mouse (dilution 1/8000, ref:A2228, Sigma-Aldrich) and Anti-Gamma tubulin, mouse (1/10000, ref: T6557, Sigma-Aldrich). After incubation with secondary antibodies for 1 h at room temperature, proteins were detected with ChemiDoc^{TM} Touch Imaging System (BioRad). The bands were quantified with ImageLab software (BioRad).

ATP measurement

Fibroblasts were plated in 96-well transparent and white plates in parallel. The transparent plates were used to monitor cell growth and for cell nuclei quantification. The white plates were used to measure luminescence. Cells were deprived of glucose for 24 h using DMEM medium without glucose (Biological Industries, ref: 01-057-1A). Then, ATP concentrations were quantified using the ATPlite 1 step assay (PerkinElmer, 6016736) according to the manufacturer's protocol in a VICTOR X5 2030 Multilabel plate reader. Experiments were performed twice, using quatriplicate wells. ATP measurements were normalized to number of cells per well, estimated by counting cell nuclei stained with DAPI.

Inner mitochondrial membrane potential quantification

The inner mitochondrial membrane potential was measured as previously described [21]. Briefly, flow cytometry was performed using the voltage-sensitive indicator, tetramethylrhodamine ethyl ester (TMRE) (Molecular Probes, T669). Fibroblasts were plated in 6-well tissue culture plates. When confluence was reached, cells were washed with PBS and incubated with 50 nM of TMRE in prewarmed PBS for 30 min at 37ºC. The cells were trypsinized, centrifuged at 1000 x g for 5 min and resuspended in prewarmed PBS. All samples were captured in a FACS CantoTM recording 20,000 cells for each condition and genotype

tested. FCCP (Sigma, C2920) (200 µM for 10 min) was used as positive control. Histograms showing the percentage of depolarized cells were obtained after gating live cells. The data were analyzed with FlowJo Tree Star software. Experiments were performed twice.

Evaluation of intracellular ROS levels

Intracellular H_2O_2 levels were estimated using the ROS-sensitive H_2DCFDA (DCF) (Thermo Fisher, D399) probe. Intracellular and mitochondrial superoxide O^{-}_2 anion levels were estimated using DHE and MitoSOX^{TM} Red probes (Thermo Fisher, D11347 and M36008), respectively, as previously described [22]. The fluorescence of the DCF-, DHE- and MitoSOX-stained cells was measured with a spectrofluorometer (FLUOstar Omega microplate reader, BMG Labtech) (excitation wavelength 493 nm, emission wavelength 527 nm for DCF; excitation wavelength 530 nm, emission wavelength 590 nm for DHE and MitoSOX). For normalization, the protein levels were measured using a Pierce BCA Protein Assay protocol (Thermo Fisher Scientific). Antimycin A (Sigma, A8674) (Ant A at 200 µM for 1 h) was used as positive control. Experiments were performed twice.

Oxygen consumption measurements

The oxygen consumption rate (OCR) of the cells was measured using a Seahorse XFe96 Extracellular Flux Analyzer (Agilent Technologies) as previously described [23]. Briefly, fibroblasts were seeded into gelatin-coated cell culture microplates at a density of 70,000 per well and allowed to adhere for 24 h. Prior to the assay, the cell culture medium was replaced with XF DMEM medium (pH=7.4) containing 2 mM glutamine, 1 mM pyruvate, 5 mM HEPES and 1 mM glucose. The OCRs were measured after sequential injections of oligomycin (1µM), FCCP (2µM) and rotenone and antimycin A (1µM). For normalization, Hoechst 33342 stain was coinjected with rotenone and antimycin A at a concentration of 20 µM. The stained nuclei were counted using Cytation1 (BioTek) and used to normalize the OCRs. The average values (±SD) of the OCRs for basal respiration, maximal respiration, spare capacity and ATP coupling were calculated using the Mitochondrial Stress Test Export Function of the Wave software. The values are expressed as the mean±SD and statistical analysis was performed using Prism 8. Experiments were performed twice.

***Drosophila* lines and Motor function assay**

Fly stocks and crosses were maintained on standard cornmeal agar mediate at 29° C. OK371-gal4 was acquired from Bloomington Stock Center and the Shmt2 (RNAi #1, ID: 19208; RNAi #2, ID: 19206) was acquired from Vienna *Drosophila* RNAi Center.OK371-gal4 (a motor neuron specific driver) was used to express Shmt2 RNAi or eGFP (control), and climbing assays were performed as previously described [1, 7]. The climbing distance (cm) and velocity (cm/s) of each fly were quantified and analyzed using GraphPad Prism 6 software. Three experimental replicates were performed for each group.

Larval preparation, immunohistochemistry and quantification

For an analysis of neuromuscular junctions (NMJ), eGFP and Shmt2 (RNAi #1 and RNAi #2) wandering third instar larvae were dissected, fixed, and immunostained [7]. Briefly, the larvae were dissected in PBS and fixed in 4% paraformaldehyde in PBS for 20 min at room temperature, washed 3 times with 0.1% PBST (0.1% Triton X-100 in PBS), and then blocked with 5% normal goat serum (Abcam; AB7681) in 0.1% PBST. Larvae were then probed overnight with primary antibody mouse anti-DLG (DSHB, 4F3, 1:100) at 4°C. The larvae were then washed three times in 0.1 % PBST and incubated in secondary antibodies (anti-mouse Alexa Flour 647, Invitrogen, # 28181, 1:100) and Cy3-conjugated anti-HRP (Jackson ImmunoResearch, Cat#:123-165-021, 1:200) for 2 h, washed three times in 0.1% PBST, and mounted using DAPI Fluoroshield (Sigma, #F6182). Images were captured using Nikon A1

eclipse Ti confocal microscope. For the analysis, NMJs from muscle 4 on segment A2-A3 were imaged from 4-5 larvae, and then, the synaptic boutons were quantified using ImageJ software (NIH). Boutons included in a chain of two or more boutons were considered mature, while single boutons that were not included in a chain and were sprouted off of a mature bouton or branch were considered as satellite boutons. Statistical analysis was performed with Prism 6 (GraphPad Software).

REFERENCES

1. Anderson EN, Gochenaur L, Singh A, et al. (2018) Traumatic injury induces stress granule formation and enhances motor dysfunctions in ALS/FTD models. Hum Mol Genet 27:1366–1381. doi: 10.1093/hmg/ddy047
2. Auton A, Abecasis GR, Altshuler DM, et al. (2015) A global reference for human genetic variation. Nature 526:68–74
3. Berman HM, Westbrook J, Feng Z, et al. (2000) The Protein Data Bank
4. Biasini M, Bienert S, Waterhouse A, et al. (2014) SWISS-MODEL: Modelling protein tertiary and quaternary structure using evolutionary information. Nucleic Acids Res 42. doi: 10.1093/nar/gku340
5. Cao J, Sun L, Aramsangtienchai P, et al. (2019) HDAC11 regulates type I interferon signaling through defatty-acylation of SHMT2. Proc Natl Acad Sci U S A 116:5487–5492. doi: 10.1073/pnas.1815365116
6. Casado M, Sierra C, Batllori M, et al. (2018) A targeted metabolomic procedure for amino acid analysis in different biological specimens by ultra-high-performance liquid chromatography-tandem mass spectrometry. Metabolomics 14:76. doi: 10.1007/s11306-018-1374-4
7. Casci I, Krishnamurthy K, Kour S, et al. (2019) Muscleblind acts as a modifier of FUS toxicity by modulating stress granule dynamics and SMN localization. Nat Commun 10:5583. doi: 10.1038/s41467-019-13383-z
8. Dhamija R, Graham JM, Smaoui N, et al. (2014) Novel de novo SPOCK1 mutation in a proband with developmental delay, microcephaly and agenesis of corpus callosum. Eur J Med Genet 57:181–184. doi: 10.1016/j.ejmg.2014.02.009
9. Ducker GS, Ghergurovich JM, Mainolfi N, et al. (2017) Human SHMT inhibitors reveal defective glycine import as a targetable metabolic vulnerability of diffuse large B-cell lymphoma. Proc Natl Acad Sci 114:11404–11409. doi: 10.1073/pnas.1706617114
10. Eddy SR (2009) A new generation of homology search tools based on probabilistic inference. Genome Inform 23:205–211. doi: 10.1142/9781848165632_0019
11. Feng Y, Kloczkowski A, Jernigan RL (2007) Four-body contact potentials derived from two protein datasets to discriminate native structures from decoys. Proteins Struct Funct Genet 68:57–66. doi: 10.1002/prot.21362
12. Giardina G, Brunotti P, Fiascarelli A, et al. (2015) How pyridoxal 5'-phosphate differentially regulates human cytosolic and mitochondrial serine hydroxymethyltransferase oligomeric state. FEBS J 282:1225–41. doi: 10.1111/febs.13211
13. Gupta A, Zimmermann MT, Wang H, et al. (2019) Molecular characterization of known and novel ACVR1 variants in phenotypes of aberrant ossification. Am J Med Genet A 179:1764–1777. doi: 10.1002/ajmg.a.61274
14. Huang J, Mackerell AD (2013) CHARMM36 all-atom additive protein force field: Validation based on comparison to NMR data. J Comput Chem 34:2135–2145. doi: 10.1002/jcc.23354
15. Jenik M, Parra RG, Radusky LG, et al. (2012) Protein frustratometer: a tool to localize energetic frustration in protein molecules. Nucleic Acids Res 40:W348-51. doi: 10.1093/nar/gks447
16. Karamzadeh R, Karimi-Jafari MH, Sharifi-Zarchi A, et al. (2017) Machine Learning and Network Analysis of Molecular Dynamics Trajectories Reveal Two Chains of Red/Ox-specific Residue Interactions in Human Protein Disulfide Isomerase. Sci Rep 7:3666. doi: 10.1038/s41598-017-03966-5
17. Karczewski KJ, Francioli LC, Tiao G, et al. (2020) The mutational constraint spectrum quantified from variation in 141,456 humans. Nature 581:434–443. doi: 10.1038/s41586-020-2308-7
18. Kocher JPA, Quest DJ, Duffy P, et al. (2014) The Biological Reference Repository (BioR): A rapid and flexible system for genomics annotation. Bioinformatics 30:1920–1922. doi: 10.1093/bioinformatics/btu137

19. Lek M, Karczewski KJ, Minikel E V., et al. (2016) Analysis of protein-coding genetic variation in 60,706 humans. Nature 536:285–291. doi: 10.1038/nature19057
20. Lek M, Karczewski KJ, Minikel E V., et al. (2016) Analysis of protein-coding genetic variation in 60,706 humans. Nature 536:285–291. doi: 10.1038/nature19057
21. Ló Pez-Erauskin J, Galino J, Bianchi P, et al. Oxidative stress modulates mitochondrial failure and cyclophilin D function in X-linked adrenoleukodystrophy. A J Neurol. doi: 10.1093/brain/aws292
22. López-Erauskin J, Galino J, Ruiz M, et al. (2013) Impaired mitochondrial oxidative phosphorylation in the peroxisomal disease X-linked adrenoleukodystrophy. Hum Mol Genet 22:3296–3305. doi: 10.1093/hmg/ddt186
23. Ma H, Folmes CDL, Wu J, et al. (2015) Metabolic rescue in pluripotent cells from patients with mtDNA disease. Nature 524:234–238. doi: 10.1038/nature14546
24. Ormazabal A, García-Cazorla A, Pérez-Dueñas B, et al. (2006) Determination of 5-methyltetrahydrofolate in cerebrospinal fluid of paediatric patients: Reference values for a paediatric population. Clin Chim Acta 371:159–162. doi: 10.1016/j.cca.2006.03.004
25. Pearl J, Causality ; M H, Kalisch M, et al. (2010) marloes h maathuis A method and server for predicting damaging missense mutations. Cambridge Univ. Press
26. Röll S, Seul J, Paulsson M, Hartmann U (2006) Testican-1 is dispensable for mouse development. Matrix Biol 25:373–381. doi: 10.1016/j.matbio.2006.05.004
27. Schwarz JM, Cooper DN, Schuelke M, Seelow D (2014) Mutationtaster2: Mutation prediction for the deep-sequencing age. Nat. Methods 11:361–362
28. Schymkowitz J, Borg J, Stricher F, et al. (2005) The FoldX web server: an online force field. Nucleic Acids Res 33:W382–W388. doi: 10.1093/nar/gki387
29. Sim N-L, Kumar P, Hu J, et al. SIFT web server: predicting effects of amino acid substitutions on proteins. doi: 10.1093/nar/gks539
30. Zimmermann MT, Urrutia R, Cousin MA, et al. (2018) Assessing Human Genetic Variations in Glucose Transporter SLC2A10 and Their Role in Altering Structural and Functional Properties. Front Genet 9:276. doi: 10.3389/fgene.2018.00276
31. Zimmermann MT, Urrutia R, Oliver GR, et al. (2017) Molecular modeling and molecular dynamic simulation of the effects of variants in the TGFBR2 kinase domain as a paradigm for interpretation of variants obtained by next generation sequencing. PLoS One 12:e0170822. doi: 10.1371/journal.pone.0170822

PATIENT DESCRIPTIONS

Patients 1 and 2 (Family 1) were born to non-consanguineous parents of Caucasian origin and no family history of genetic disease was found. Patient 1 was born at 40 weeks gestation with microcephaly (occipitofrontal circumference (OFC), 32.3 cm; -2.1 SD). He presented neonatal hypotonia and global developmental delay; his first words were spoken at 2 years of age and autonomous deambulation was established at 3.5 years. Hypertonia of the legs associated with brisk deep reflexes, Babinski sign, and bilateral clonus was detected at 4 years of age. Hypertrophic cardiomyopathy was found at 7 years of age. Currently, the patient is 10 years old and has an ejection fraction (EF) of 65%. His cranial perimeter is 51.5 cm (-2.1 SD) and his equivalent age is 21 months according to the Vineland test. He is able to walk alone with spastic abduction-inner rotation pattern gait. Axonal neuropathy has been recently detected. His older brother Patient 2 was born at 40 weeks of gestation with microcephaly (-2.4 SD). He presented axial hypotonia and appendicular hypertonia with hyperreflexia since the neonatal period and global developmental delay. His first words were spoken at 28 months of age and autonomous deambulation was established at 3.5 years. He did not have history of clinical seizures, but at 4 years of age, electroencephalogram revealed right temporal beta activity during sleep that suggested a cortical dysplasia. At age of 14 years a distal axonal neuropathy was diagnosed, that tended to progress over time and an hypertrophic cardiomyopathy without left ventricular outflow tract obstruction (LVOT) and mild reduction in EF was detected at 17 years of age in the context of an infectious respiratory event. Currently, he suffers from severe spastic paraparesis and uses an electric wheelchair at 20 years of age. Also, echocardiogram revealed a worsening of left ventricular EF of 40%**.**

Patient 3 (Family 2) is a 19-year-old girl, of Caucasian non-consanguineous parents. The mother previously had a male fetus (terminated at 21 weeks) with severe microcephaly. The patient showed intrauterine growth restriction detected at 35 weeks gestation and was born with microcephaly and a small atrial septal defect. Later, she was found to have an aberrant right subclavian artery and vascular ring. She has global developmental delay, thin corpus callosum, appendicular hypertonia with hyperreflexia, axial hypotonia, dysarthria, dystonia, intention tremor, and poor fine motor control. At 19 years of age, her cranial perimeter is in the ~25th percentile. Echocardiogram revealed mildly increased concentric left ventricular wall thickness, an EF of 67%, and the averaged peak left ventricular global longitudinal strain was borderline at -18 % at 19 years of age.

Patient 4 (Family 3) is a female, first child of healthy Caucasian non-consanguineous parents, born at 40 weeks of gestation with microcephaly (31.5 cm; -2.3 SD), which had been detected during the third trimester of pregnancy. She said her first words and walked independently at 14 months and 24 months of age, respectively. At 2 years of age, she developed intention hand tremor, which was more marked at the left side, and pyramidal signs in the lower extremities (hypertonia, brisk deep tendon reflexes, and Babinski sign). Hypertrophic cardiomyopathy was detected at 9 years of age in the context of an infectious respiratory event. Severe dysfunction of the left ventricle (EF: 35%) led to heart transplantation some months later because of a rapid worsening (EF: 21%). The child is currently 12 years old, with microcephaly below the first percentile (-2.6 SD) and has moderate intellectual disability. She has a paraparetic genu-flexum gait but can walk long distances without help, mild intention tremor and dysmetria.

Patient 5 (Family 4) is a 2-year-old boy, the second child of healthy Caucasian non-consanguineous parents. Microcephaly in the 3rd centile was diagnosed prenatally, and a fetal cerebral MRI showed a two-week delay in maturation. He was born at 38 weeks of gestation with microcephaly (OFC <3rd centile). He had early feeding difficulties, which was remediated by a nasogastric tube during the first weeks. Microretrognathism, bivid uvula, long palpebral fissures and overlapping sutures were present at birth. Cerebral computed tomography ruled out craniosynostosis. Echocardiography showed an atrial septal defect and ostium secundum. He has a severe global developmental delay, first holding his head up at 12 months, and unable to

sit or speak at 2.5 years of age. He had no facial movement and showed hypersalivation, suggesting facial diplegia. He fails to thrive, with a weight at 2.5 SD, a height at -2 SD, and microcephaly with OFC at -3 SD.

Individual	F3 (P4)	F4 (P5)	F2 (P3)	F4 (P5)	F2 (P3)	F3 (P4)	F1 (P1, P2)
**Genomic position (hg19)**	Chr12:57625653 C>T	Chr12:57626038 C>G	Chr12:57627528_ 57627533 delinsA	Chr12:57627543 A>G	Chr12:57627675 G>A	Chr12:57627810 A>C	Chr12:57628124 C>G
**Genomic position (hg38)**	Chr12:57231870 C>T	Chr12:57232255 C>G	Chr12:57233745_ 57233750delinsA	Chr12:57233760 A>G	Chr12:57233892 G>A	Chr12:57234027 A>C	Chr12:57234341 C>G
**cDNA change (NM_005412.6)**	c.469 C>T	c.557C>G	c.1124-4_1125delinsA	c.1135A>G	c.1267G>A	c.1304A>C	c.1495C>G
**RNA change (NM_005412.6)**	NA	NA	r.1124_1126del	NA	NA	NA	NA
**Protein change (NP_005403.2)**	p.Pro157Ser	p.Thr186Arg	p.Gly375del	p.Asn379Asp	p.Gly423Ser	p.Gln435Pro	p.Pro499Ala
**Frequency in gnomAD v2.1.1 (#/total alleles)**	1/248,762	0/251,460	0/282,384	0/251,096	6/251,332	0/251,486	0/241,364
**Frequency in gnomAD v3 (#/total alleles)**	0/143,344	0/143,306	0/143,340	0/143,362	4/143,334	0/143,254	0/143,264
**SIFT (score)**	Tolerated (0.4)	Deleterious (0.01)	NA	Tolerated (0.14)	Deleterious (0)	Tolerated (score: 0.18)	Deleterious (0)
**Mutation Taster (probability)**	Disease causing (1)	Disease causing (1)	NA	Disease causing (1)	Disease causing (1)	Disease causing (1)	Disease causing (1)
**PolyPhen-2 (score)**	Probably damaging (1.000)	Probably damaging (1.000)	NA	Possibly damaging (0.564)	Probably damaging (1.000)	Benign (0.098)	Probably damaging (1.000)
**M-CAP (score)**	Possibly pathogenic (0.026)	Possibly pathogenic (0.032)	NA	Possibly pathogenic (0.028)	Possibly pathogenic (0.417)	Likely benign (0.018)	Possibly pathogenic (0.049)
**PredictSNP2 (% expected accuracy)**	Deleterious (87%)	Deleterious (87%)	NA	Deleterious (87%)	Deleterious (87%)	Deleterious (82%)	Deleterious (87%)

Supplementary Table 1: Features of SHMT2 variants identified in patients

**Protein Variant**	**ΔΔG_{fold} (Kcal/mol)**	**ΔSASA (Å2)**	**ΔFour-body Potential**	**ΔSRLF**
**p.Pro157Ser**	6.23	-10.93	-5.22	0.11
**p.Thr186Arg**	10.23	-4.32	12.95	-0.03
p.Gly375Ala (used in analogy to **p.Gly375del**)*	3.23	24.70	-4.41	1.14
**p.Asn379Asp**	2.79	-0.65	0.76	-0.11
**p.Gly423Ser**	6.64	-0.22	-11.70	-0.37
**p.Gln435Pro**	12.81	-33.99	-0.66	0.35
**p.Pro499Ala**	5.82	0.16	1.88	1.14
p.Arg437His (benign variant)	1.48552	-42.66	1.92	-0.91
p.Ser50Leu (benign variant)	-0.989257	38.71	-3.48	0.03

Supplementary Table 2: Predictors of structural integrity for SHMT2 variants' 3D models. SHMT2 variants are all predicted to highly destabilize SHMT2 structure. ΔΔGfold and ΔFour-body potential values measure predicted protein stability. Solvent Accessible Surface Area (SASA) values are a measure of the protein's total surface area. Changes in SASA values can indicate changes in the protein's conformation. Single Residue Level Frustration (SRLF) measures the balance (or imbalance) between favorable and un-favorable contacts within the protein structure. Increases in SRLF may indicate protein instability. * p.Gly375Ala is observed rarely in gnomAD (MAF=1.45x10-5). We used it to evaluate p.Gly375del, but under the expectation that it will not be as severe. Still, alanine has a less flexible backbone compared to glycine, and results are supportive of the need for glycine at this position.

Protein Variant	ΔPC1 † (σ)	ΔPC2 † (σ)	Dynamics-Based interpretation[γ]
**p.Pro157Ser**	0.8	0.1	Impaired oligomerization. Shift in outer helices that is symmetric by the dimer
**p.Thr186Arg**	0.6	-0.6	Local instability; monomers move apart by Arg186
**p.Gly375del**	-0.6	0.3	Altered active site shape. Indirectly affects the ligand interaction pocket
**p.Asn379Asp**	-0.6	0.4	Altered active site making it more spacious
**p.Gly423Ser**	0.1	1.5	Altered active site gating
**p.Gln435Pro**	0.7	-0.8	Local instability
**p.Pro499Ala**	1.2	-0.2	Altered active site shape, Local instability; dimer interface alteration

Supplementary Table 3: Principal Component analysis variations for 3D dynamics simulations in mutated SHMT2 models. PC1 indicates a motion of SHMT2 wherein the outer helices close in towards to active site, while PC2 corresponds to opening of one monomer's active site concurrent to remodeling of the dimer interface. † Differences in PC sampling from WT, expressed as the shift in the number of median-based standard deviations from the WT median. We combined structure- and dynamics-based scores into a composite prediction of effect.

	Family F1		Family F2	Family F3	Family F4
	**PATIENT 1**	**PATIENT 2**	**PATIENT 3**	**PATIENT 4**	**PATIENT 5**
**Ethnicity**	Caucasian	Caucasian	Caucasian	Caucasian	Caucasian
**Onset/Current Age**	Prenatal/10 years	Neonatal/20 years	Prenatal/19 years	Prenatal/12 years	Prenatal/ 2 years

**PERINATAL PERIOD**					
**Growth Parameters**	W: 3630 gr (0.7 SD); HC: 32.3 cm (-2.1 SD) 51.5 cm (-2.1 SD) at last investigation	W: 3320 gr (0.02 SD); HC: 32 cm (-2.4 SD) 54.5 cm (1.3 SD) at last investigation	W: 2578 gr (-1.5 SD); HC: 28.5 cm (-3 SD) 53.5 cm (-1.5 SD) at last investigation	W: 2840 gr (0.8 SD); HC: 31.5 cm (-2.3 SD) 51 cm (-2.43 SD) at last investigation	W: 2700 gr (-1.4 SD); HC: 31.5 cm (-2.9 SD) 46cm (-3SD) at last investigation
**Neurological signs**	Microcephaly detected during pregnancy. Hypotonia at birth	Microcephaly at birth Hypertonia of the extremities	Microcephaly detected during pregnancy.	Microcephaly detected during pregnancy	Microcephaly detected during pregnancy
**Developmental Skills**	Says first words at 2 years of age, walks at 3.5 years of age.	Says first words at 28 months of age, walks at 3.5 years of age.	Sits at 9 months of age, crawls at 17 months and walks at around 26 months of age.	Holds her head steady at 4 months of age, sits unsupported at 8 m, first words at 14 months, walks at 24 months of age.	Head holding at 12 months, no sitting, no word

**COGNITION AND BEHAVIOUR (current age)**					
**Psychometric test**	Vineland Adaptative Behavior. Equivalent age: 21 months. Severe intellectual disability.	Vineland Adaptative Behavior. Equivalent age: 22 months. Severe intellectual disability.	Not performed	WISC-V: moderate intellectual disability	Not performed
**Adaptative behavior**	SS 52	SS 29	Clinically estimated: mild-moderate intellectual disability	Total IQ. 42 Verbal IQ: 50 Visuospatial IQ: 53	Probably severe ID
**Communication**	SS 53	SS 33			Not evaluable
**Daily living skills**	SS 55	SS 28			Not evaluable
**Socialization**	SS 58	SS 37			Not evaluable
**Anxiety-OCD**	Yes, anxiety	Yes, anxiety	Yes, anxiety	No	Not evaluable
**ASD-ADHD**	ADHD-like	Hand stereotypies, autistic traits and short attention span	ADHD-like	ADHD-like	Not evaluable
**Others**		Agressive bursts		Dysfunctional adaptative behavior	

MOTOR SYMPTOMS					
**Spasticity**	At the lower limbs, with Increased deep tendon reflexes and extensor reflex	At the lower limbs, with Increased deep tendon reflexes and extensor reflex. Scissors gait.	Hypertonia and hyperreflexia of the limbs	Hypertonia and hyperreflexia of the limbs, spastic gait.	Spastic tetraplegia
**Ataxia/Dysmetria**	No	Truncal ataxia, Spastic-Dyspraxic gait. Dysmetria	Dysmetria	Spastic-Dyspraxic gait. Dysmetria. SARA scale: 5 points	Not evaluable
**Others**		Intention tremor.<br>Hand Stereotypies	Intention Tremor.<br>Dystonicmovements.	Intention tremor. Mild hypokinesia and bradykinesia	
**Peripheral neuropathy**	Distal neuroaxonal impairment.	Progressive axonal and sensory neuropathy	No studies performed	Nerve conduction study was normal at 9 years of age.	No studies performed
**Brain MRI**	Corpus callosum hypoplasia. Bilateral Perisylvian polymicrogyria	Corpus callosum hypoplasia. Bilateral Perisylvian Polymicrogyria	Hypoplasia and dysmorphy of the corpus callosum. Unilateral perisylvian polymicrogyria	Corpus callosum hypoplasia. Unilateral perisylvian polymicrogyria	Thin corpus callosum, ventriculomegaly

OTHER FEATURES					
**Cardiac symptoms**	Hypertrophic Cardiomyopathy (at 5 years of age)	Hypertrophic Cardiomyopathy with left moderate ventricular dysfunction, ejection fraction of 40%(at 16 years of age)	Hypertrophic Cardiomyopathy (at 3 years of age)	Severe hypertrophic cardiomyopathy detected at 9 years of age leading to heart transplantation	Atrial Septum Defects, ostium secondum.
**Dysmorphology**	Arched eyebrows, thin upper lip, long philtrum, Low-set thumbs. 2-3 toe syndactyly	Arched eyebrows, low-set thumbs. 2-3 toe syndactyly	Short philtrum, small downturned mouth Digitalized thumbs	Arched eyebrows, thin upper lip,low-set thumbs. 2-3 toe syndactyly	Facial diplegia, retrognathia, bifid uvula. closed hands. Adductus thumbs.
**Other clinical features**	Conjunctival hyperemia Ocular movements: slow saccades. Drooling. Orofacial dyspraxia	Conjunctival hyperemia Scoliosis. Ocular movements: slow saccades. Drooling. Orofacial dyspraxia EEG bifrontal discharges without clinical seizures	Subclavian vascular ring. Cyclic vomiting. Strabismus of right eye, tonic upward eye movement, mild amblyopia	Expressive language disorder	Mild scoliosis. Hip dislocation. Divergent strabismus.

Supplementary Table 4: Clinical features of SHMT2-mutated patients. ADHD: Attention deficit hyperactivity disorder; ASD: Autism spectrum disorder; HC: Head Circumference; N: Normal values; SS (Standard Score): 100+15; W: Weight.

	Control 1	Control 2	Control 3	Control 4	Control 5	Control 6	Patient 1	Patient 2	Patient 3	Patient 4	Patient 5
**Ala**	29.09	34.09	67.89	45.77	52.04	22.48	40.67	35.14	55.18	29.81	26.64
**Arg**	3.11	9.94	14.30	8.50	30.37	10.79	12.18	10.42	10.04	11.43	12.11
**Asn**	0.9	5.04	**NA**	7.48	26.76	7.57	5.92	2.52	**NA**	**NA**	10.99
**Asp**	88.74	140.14	128.76	78.11	91.47	109.19	86.95	39.58	74.29	56.88	66.77
**Cys**	0.03	0.06	0.16	0.13	0.139	0.103	0.15	0.18	0.17	**NA**	0.146
**Gln**	86.60	85.55	100.99	105.25	83.47	90.95	102.01	81.41	59.59	30.74	67.61
**Glu**	190.32	237.89	400.43	232.57	342.28	375.28	229.15	197.94	147.92	311.28	291.26
**Gly**	103.00	106.00	167.00	129.00	144.67	106.68	91.00	84.00	107.00	72.00	57.55
**His**	1.52	2.22	2.07	2.73	11.76	4.93	3.97	3.30	11.39	6.62	6.33
**Ile**	7.21	9.77	20.88	9.52	34.30	15.65	9.87	11.95	8.09	16.26	18.61
**Leu**	11.30	16.10	26.19	12.19	47.43	18.70	14.09	17.13	11.89	21.47	24.15
**Lys**	10.76	13.09	19.19	11.42	34.01	15.74	11.55	17.04	13.1	20.02	16.93
**Met**	3.50	5.70	7.70	3.30	15.77	5.98	3.04	5.90	2.70	6.10	6.93
**Orn**	4.51	4.01	4.94	6.65	4.18	3.10	7.87	7.62	22.15	14.22	3.68
**Phe**	6.60	9.33	15.31	7.74	29.43	10.82	8.72	10.46	6.23	12.30	13.48
**Pro**	34.38	35.46	86.42	48.50	50.74	37.24	40.56	38.48	32.58	30.45	29.86
**Ser**	33.40	39.10	52.40	55.80	55.48	32.78	61.50	44.40	114.00	52.00	40.83
**Thr**	33.61	40.58	60.92	45.49	72.12	47.69	47.89	36.03	35.42	32.22	46.20
**Trp**	1.95	2.53	4.21	2.32	9.04	3.43	2.54	2.92	1.87	3.85	4.27
**Tyr**	8.37	11.78	18.91	9.92	36.50	11.77	10.47	13.08	8.49	13.54	14.31
**Val**	10.07	13.42	21.83	12.11	41.16	16.18	13.94	15.29	13.98	20.67	21.60
**Gly/Ser**	3.08	2.71	3.19	2.31	2.61	3.25	1.48	1.89	0.94	1.38	1.41
**5-metTHF**	19.10	21.30	19.30	20.40	30	**NA**	26.10	26.10	31.70	28.00	39.00
**Total Folate**	132.00	149.00	144.00	132.00	136.5	**NA**	127.00	95.00	97.00	184.00	176
**5-metTHF / total folate**	0.14	0.14	0.13	0.15	0.11	**NA**	0.21	0.27	0.33	0.15	0.22
**THF (mitochondrial)**	0.00	0.00	0.00	0.00	**NA**	**NA**	0.79	1.02	0.30	0.10	**NA**

Supplementary Table 5: Quantification of amino acids (μmol/L) and folates (nmol/L) in control (n=6) and patient (n=5) fibroblasts. All fibroblast samples were prepared in the same way (1 million of cells in 200 uL of PBS). In yellow, aminoacid levels of relevant aminoacids/species for SHMT2 function.NA: Not analyzed.

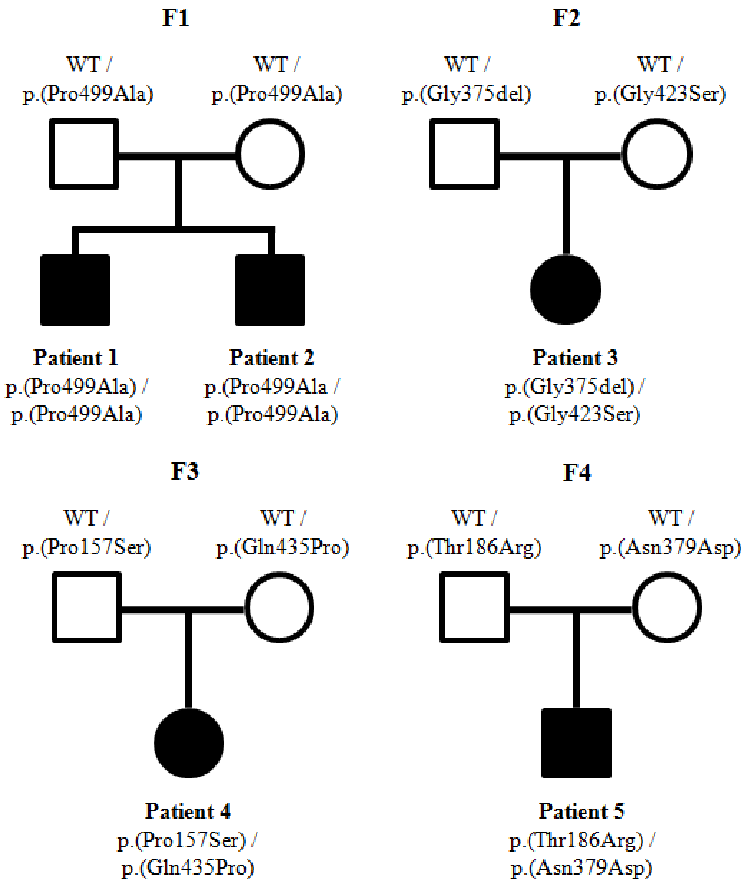

Supplementary Figure 1: Family trees from patients harboring SHMT2 biallelic variants. Square: male, circle: female, black symbols: affected individuals, white symbols: unaffected carriers, WT: wild-type allele.

	157		186		375 379		423		435		499
Mutated	LLQSHDR	…	ISARSIF	…	LVS-GTDDHLV	…	TPGSLRL	…	TSRPFRE	…	FPMAGFD
H. sapiens	LLQPHDR	…	ISATSIF	…	LVSGGTDNHLV	…	TPGGLRL	…	TSRQFRE	…	FPMPGFD
P. Troglodytes	LLQPHDR	…	ISATSIF	…	LVSGGTDNHLV	…	TPGGLRL	…	TSRQFRE	…	FPMPGFD
M. musculus	LLQPHDR	…	ISATSIF	…	LVSGGTDTHLV	…	TPGGLRL	…	TSRQFRE	…	FPMPGFD
C. livia	LLQPHDR	…	ISATSIF	…	LVSGGTDNHLV	…	TPGGLRL	…	TSRHFYE	…	FPMPGFS
X. laevis	VLQPHDR	…	ISATSIY	…	LVSGGTDNHLV	…	TPGGLRL	…	TSRNFKE	…	FPMPGFD
D. rerio	VLNPHER	…	ISATSIY	…	LVSGGTDNHLV	…	TPGGLRL	…	TSRQLKE	…	FPMPGFH
D. melanogaster	VCRPHDR	…	ISATSIF	…	VATGGTDVHLV	…	NPSGIRL	…	TTRGLAE	…	FPLPGLE

Supplementary Figure 2: SHMT2 amino acid sequence alignment across several species shows strong conservation of the mutated residues.

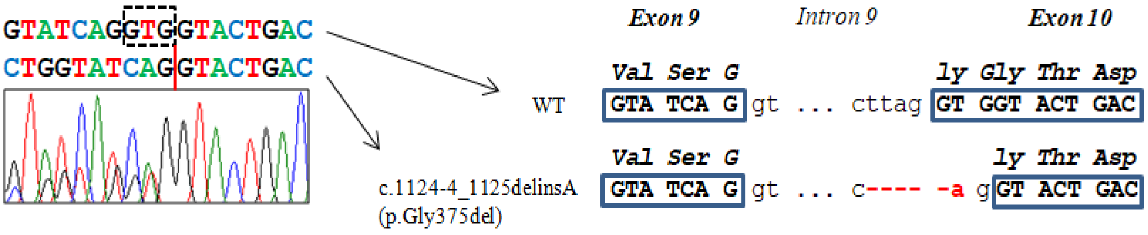

Supplementary Figure 3: Blood RNA sequencing reveals abnormal splicing from *SHMT2* indel, c.1124-4_1125deinsA, in Patient 3. Left: Sanger sequencing results of Patient P3 fibroblasts' cDNA. Right: Representation of indel variant (c.1124-4_1125delinsA) impact on splicing, leading to the creation of a novel splice acceptor, loss of three coding nucleotides of the mRNA transcript (r.1124_1126del), and deletion of a single glycine residue p.(Gly375del).

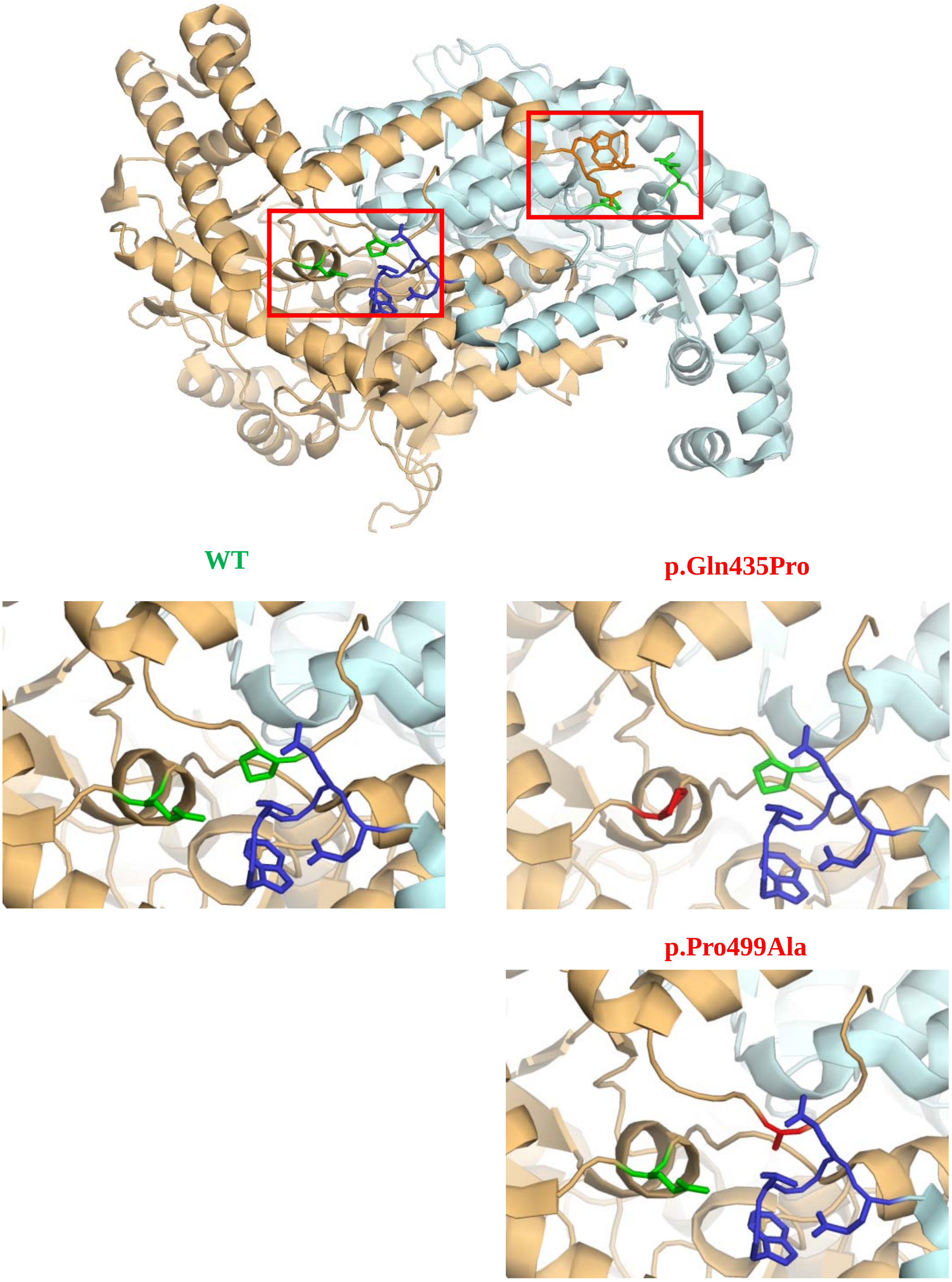

Supplementary Figure 4: Location of p.Pro499Ala and p.Gln435Pro variants in the contact interface between SHMT2 monomers. Both p.Pro499Ala and p.Gln435Pro are located very close to the N-Terminal of the opposite SHMT2 monomer in the dimeric conformation, thus impairing interactions in 2 different sites of the SHMT2 dimer. Orange and cyan correspond to different monomers of an SHMT2 dimer. Green: wild-type amino acids affected by SHMT2 mutations. Red: mutated amino acids. Dark blue, important aminoacids interacting with wild-type aminoacids (SHMT2 N-Term).

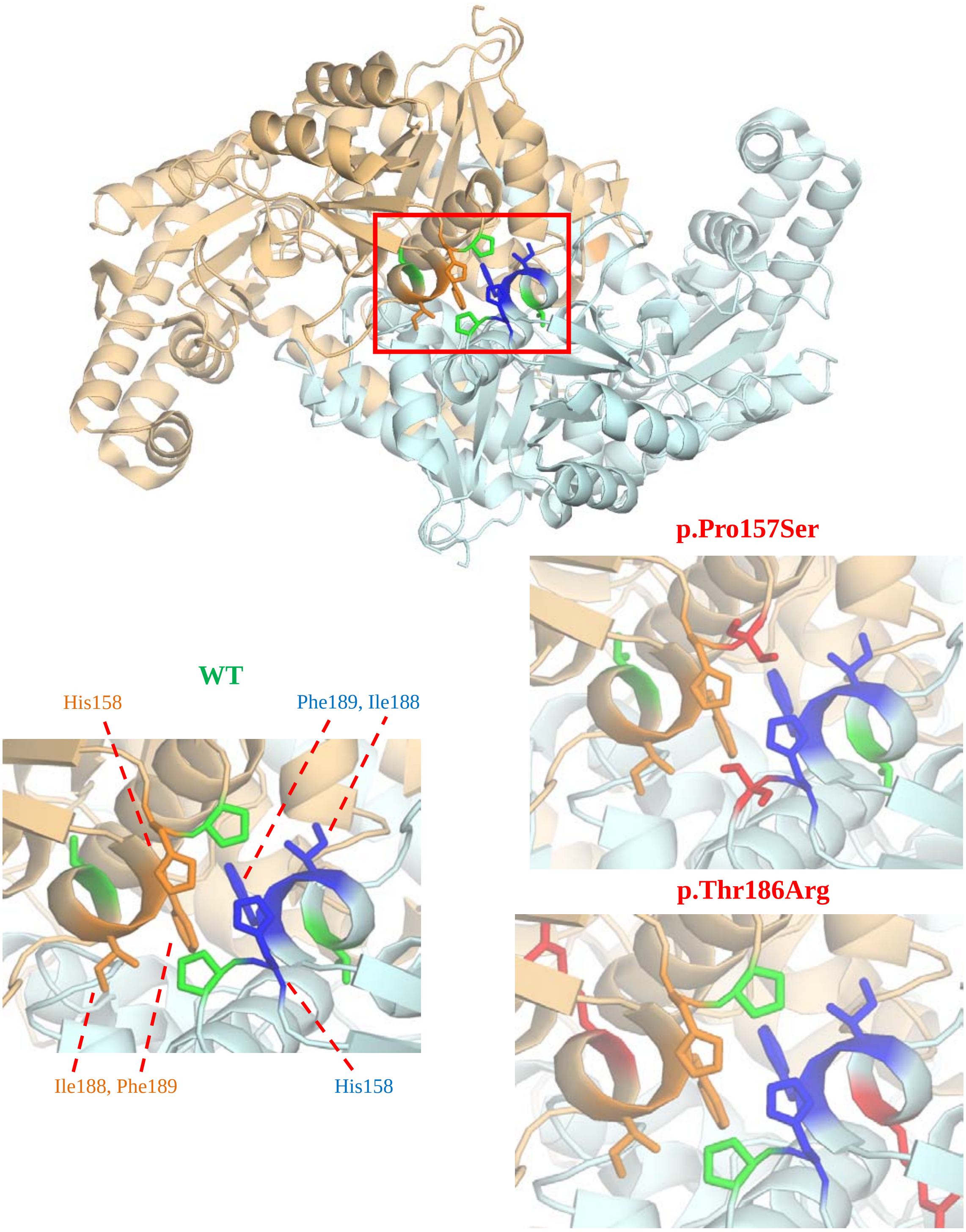

Supplementary Figure 5: Location of p.Pro157Ser and p.Thr186Arg variants in the contact interface between two SHMT2 monomers. Variants p.Pro157Ser and p.Thr186Arg probably disrupt interactions between Pro157 in one monomer and the Ile188, Phe189 amino acids in the opposite monomer; p.Pro157Ser also likely has an effect on the nearby His158 residue, which is crucial for SHMT2 tetramerization (Jagath et al., 1997). Orange and cyan correspond to different monomers of an SHMT2 dimer. Green: wild-type amino acids affected by SHMT2 mutations. Red: mutated amino acids. Dark blue and dark orange: important residues for interaction between monomers. 146-172 amino acids are important in active site pocket formation in the dimer-tetramer transition, and interaction between His158 residues belonging to 4 different SHMT2 monomers is needed for SHMT2 tetramerization.

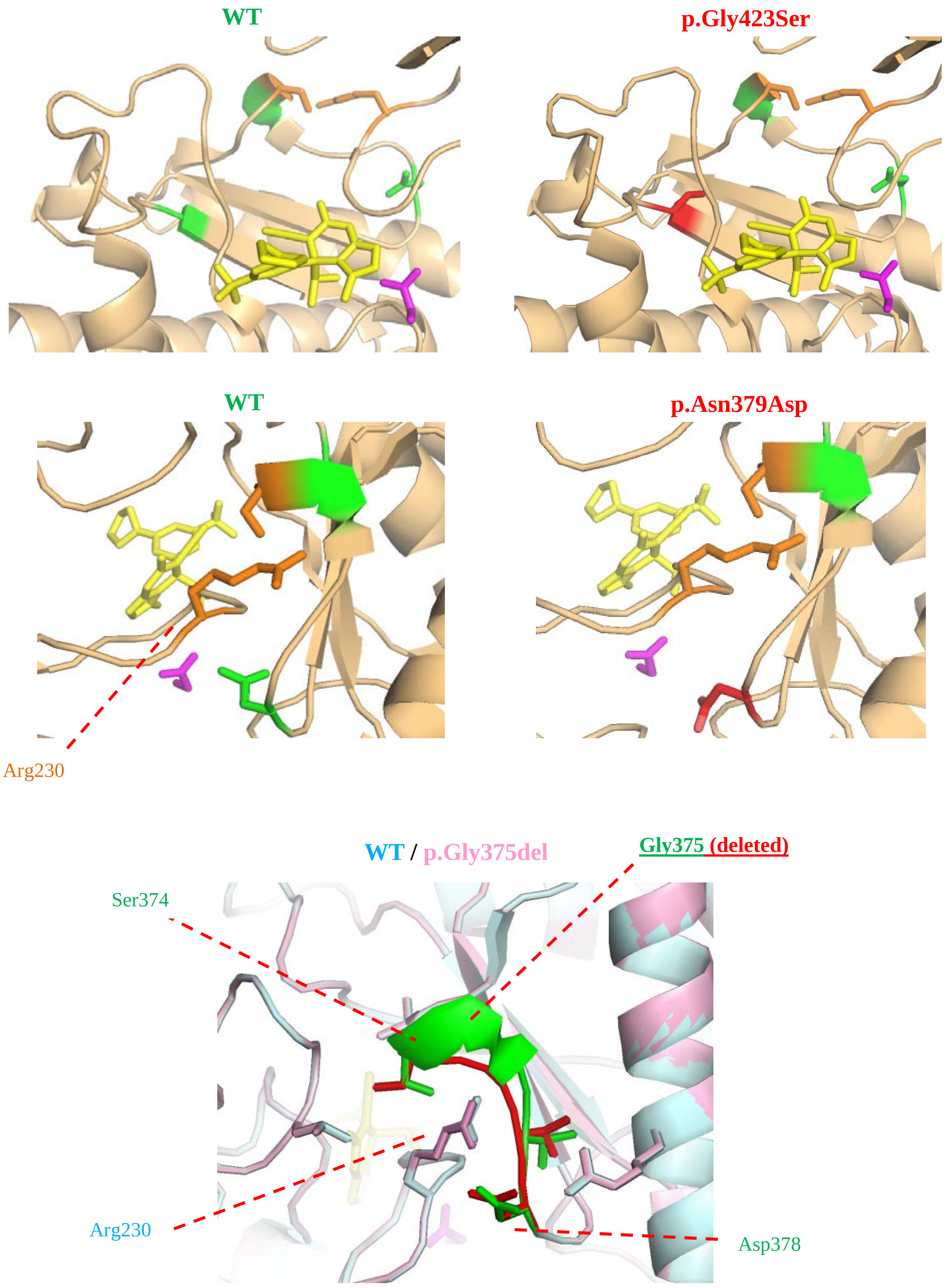
WT
p.Gly423Ser
WT
p.Asn379Asp
Arg230
WT / p.Gly375del
Gly375 (deleted)
Ser374
Arg230
Asp378

Supplementary Figure 6: Location of p.Gly375del, p.Asn379Asp and p.Gly423Ser variants at the base of loops folding over the active site. Two of these variants occur at glycine residues, causing a loss of flexibility that probably alters loop conformation and thereby move residues within the substrate-binding site away from their native positions. Similarly, p.Asn375del and p.Asn379Asp likely alter interactions with a nearby loop that is part of the active site surface. Green: wild-type amino acids affected by SHMT2 mutations. Red: mutated amino acids. Pink: Glycine (product). Yellow, SHMT2 inhibitor (*racemic compound 2*, described in Ducker et al., 2017), which occupies folate binding space.

In the case of the p.Gly375del model below (soft pink), the structure is superimposed on a cyan wild-type monomer. The deletion of Gly375 causes disruption of wild-type secondary structure, resulting into a different spatial positioning of Ser374 and Asp378, which likely interact with Arg230 in the wild-type situation. That would destabilize the loop that contains Arg230, which makes up part of the active site surface.

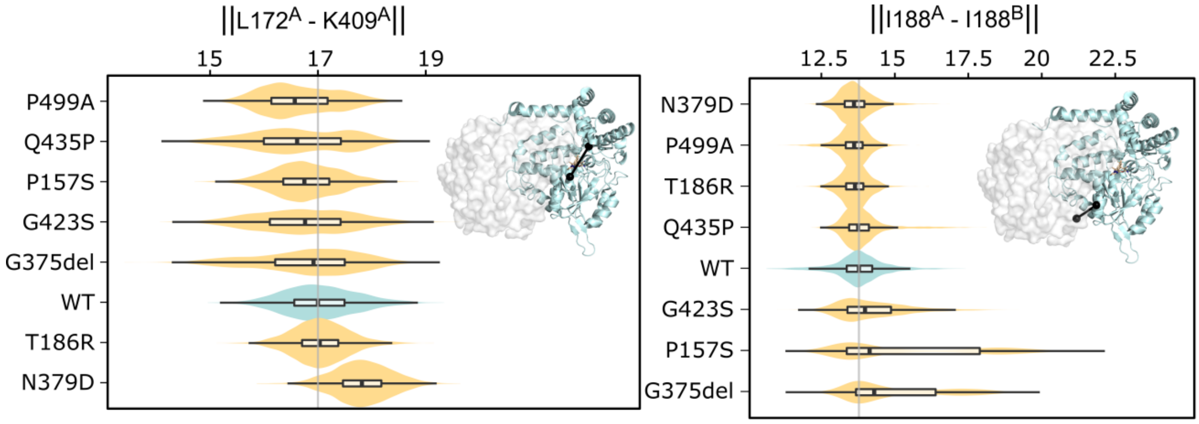

Supplementary Figure 7: Molecular dynamics simulations show that SHMT2 variants alter the binding pocket or dimer interface. Boxplots indicate the extent of sampling for each measured distance, and smoothed densities represent the amount of time each simulation was sampled across each distance value. Distance is indicated in angstroms (Å). Left: distance measured across the SHMT2 active site (Leu172-Lys409). Right: distance between 2 SHMT2 monomers (Ile188 residues).

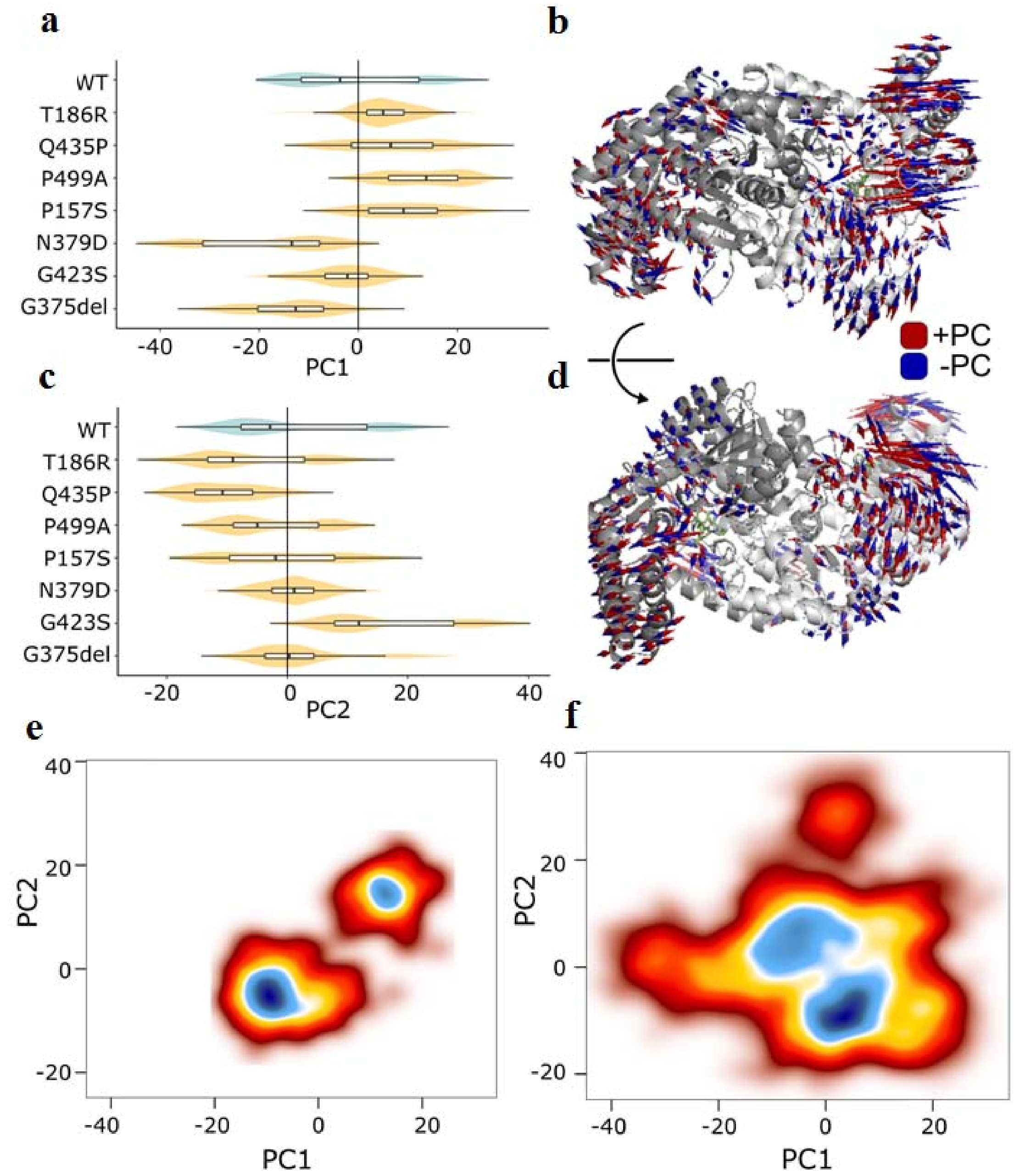

Supplementary Figure 8: Molecular dynamics 3D models of SHMT2 variants show strong alterations during conformational changes. Movements of SHMT2 models were quantified using principal components PC1 and PC2. a,c) Boxplots indicate the extent of sampling for each PC, and smoothed densities represent the duration of each simulation sampled for each PC value. b,d) Porcupine plots illustrating the motion captured by principal components PC1 (b) and PC2 (d). Positive directions are represented as spikes in red, negative in blue. Models for p.Thr186Arg, p.Gln435Pro, p.Pro499Ala and p.Pro157Ser (variants impairing likely impairing dimerization) biased towards the positive direction of PC1, indicating a more closed conformation than WT, while p.Asn379Asp, p.Gly423Ser, and p.Gly375del (variants likely altering active site) indicated a more open conformation. For PC2, p.Gly423Ser differed significantly from the wild-type model. e,f) Conformations of SHMT2 wild-type (e) mutated models (f) represented by plotting free energy landscape on PC1 and PC2 principal components. Mutated SHMT2 3D models shifted both PCs simultaneously, leading to a broader sampling, and greater regions of PC space that were not sampled in the wild-type model.

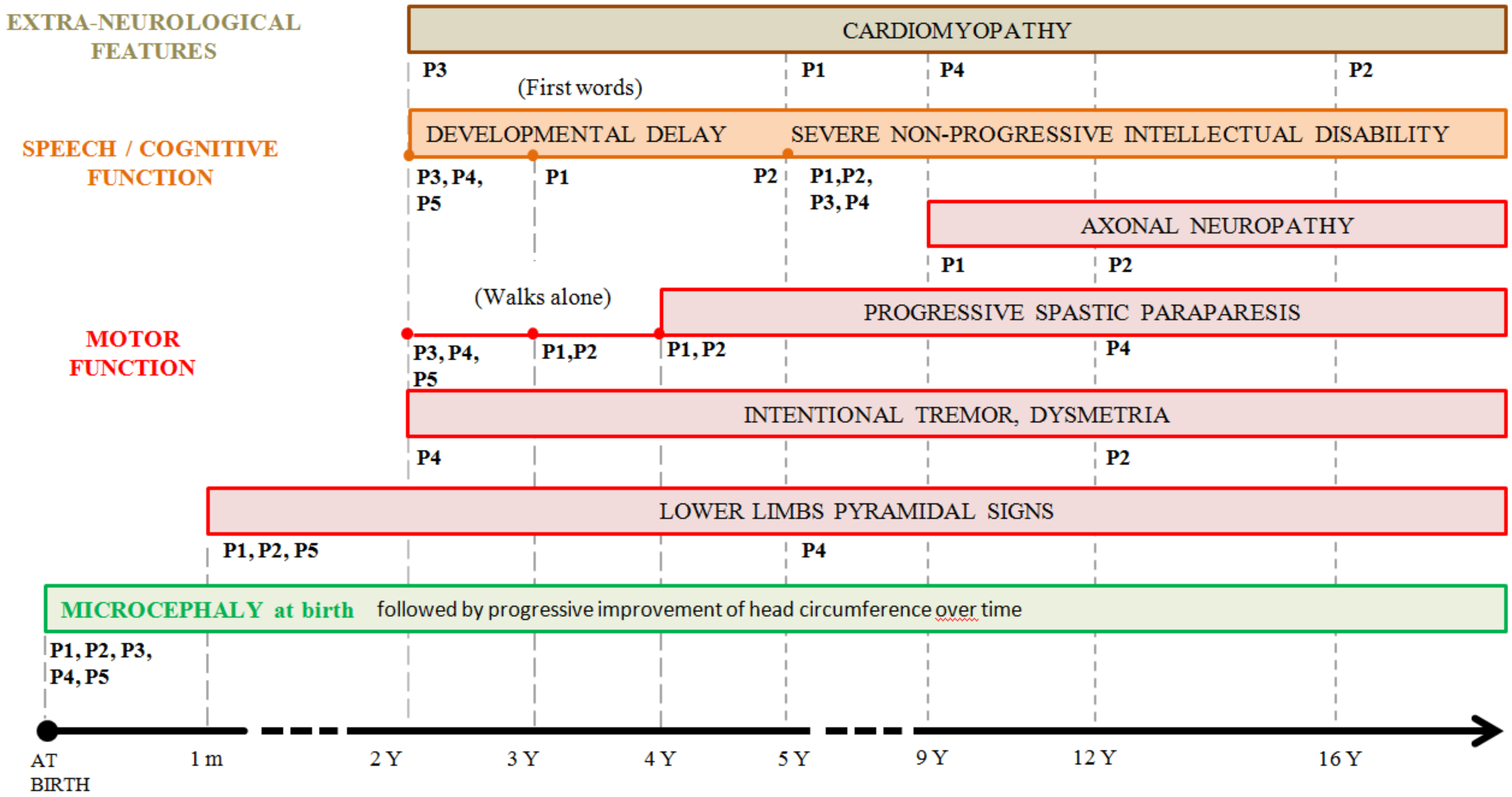

Supplementary Figure 9: Illustration showing the main clinical features and onset in patients. m: month. Y: year. P1-5: Patients.

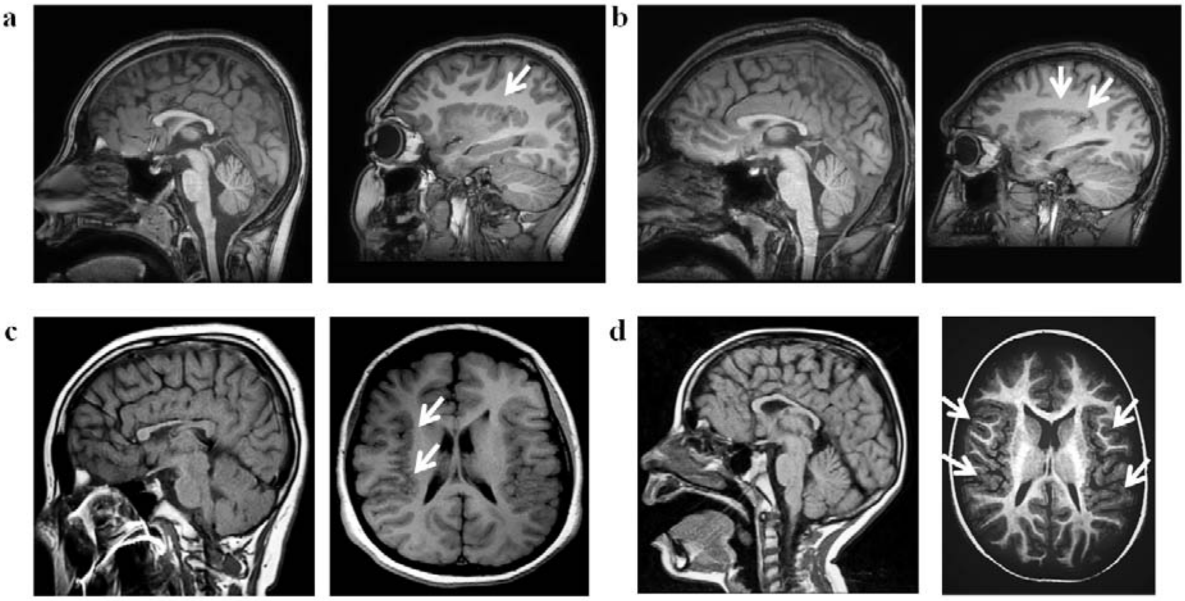

Supplementary Figure 10: MRI features of patients with biallelic SHMT2 variants. a) Patient P1; b) Patient P2; c) Patient P3; d) Patient P4. a-d: Left images: sagittal T1-weighted MRI planes. a,b: Right images: sagittal T1-weighted MRI planes. c,d: Right images: axial T1-weighted MRI planes. Patients P1 and P2 show corpus callosum hypoplasia and bilateral perisylvian polymicrogyria like-pattern. Patient P3 shows hypoplasia and dysmorphy of the corpus callosum, and perisylvian polymicrogyria-like better seen on the right side. Patient P4 shows corpus callosum hypoplasia and bilateral perisylvian polymicrogyria-like pattern. White arrows indicate polymicrogyria sites.

a

b

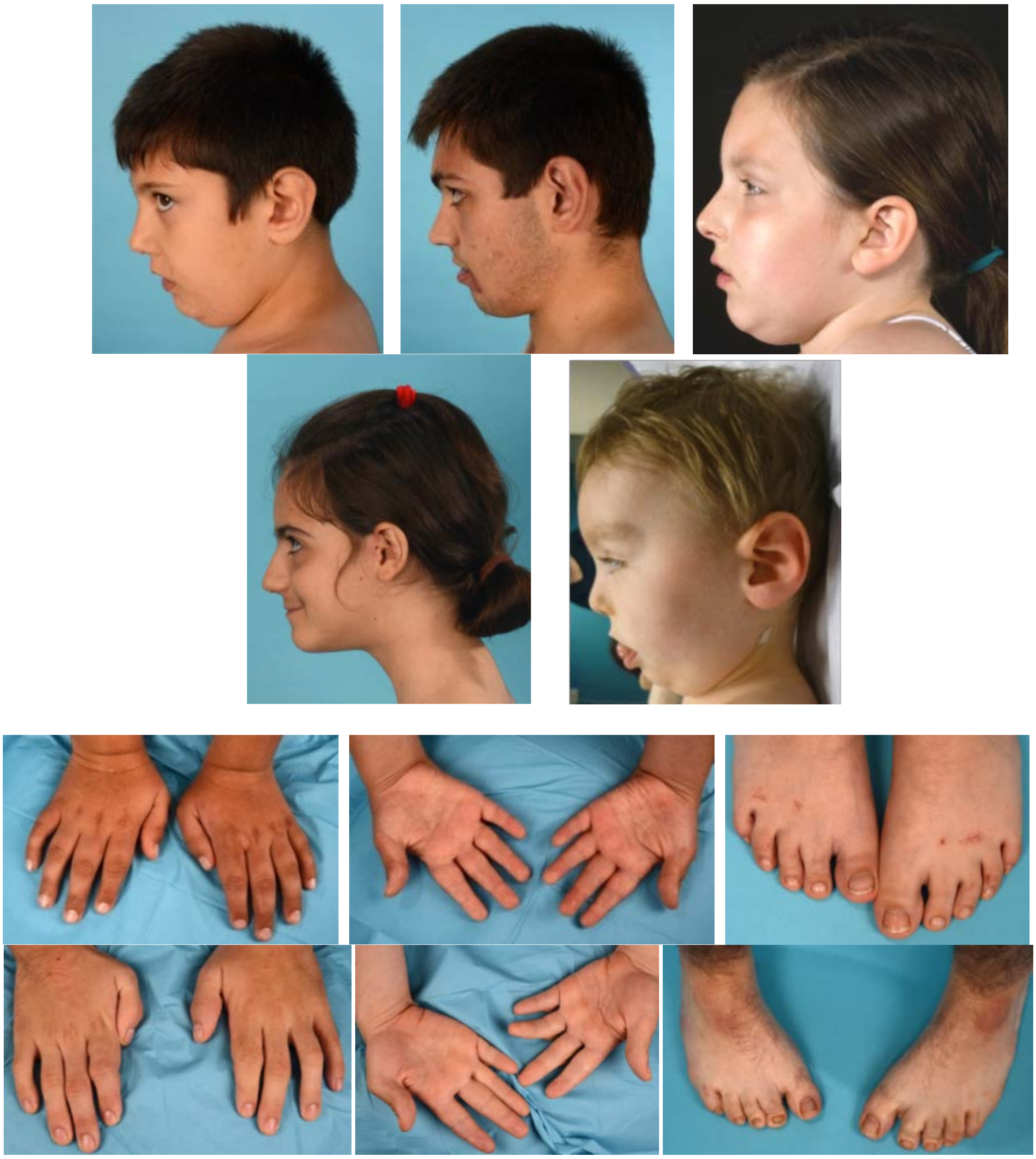

Supplementary Figure 11: Dysmorphic features in SHMT2 Patients. a) Lateral photographs of patients P1-P5 (in order, from left to right). b) Hand and feet abnormalities in Patient 1(above) and Patient 2 (Below).

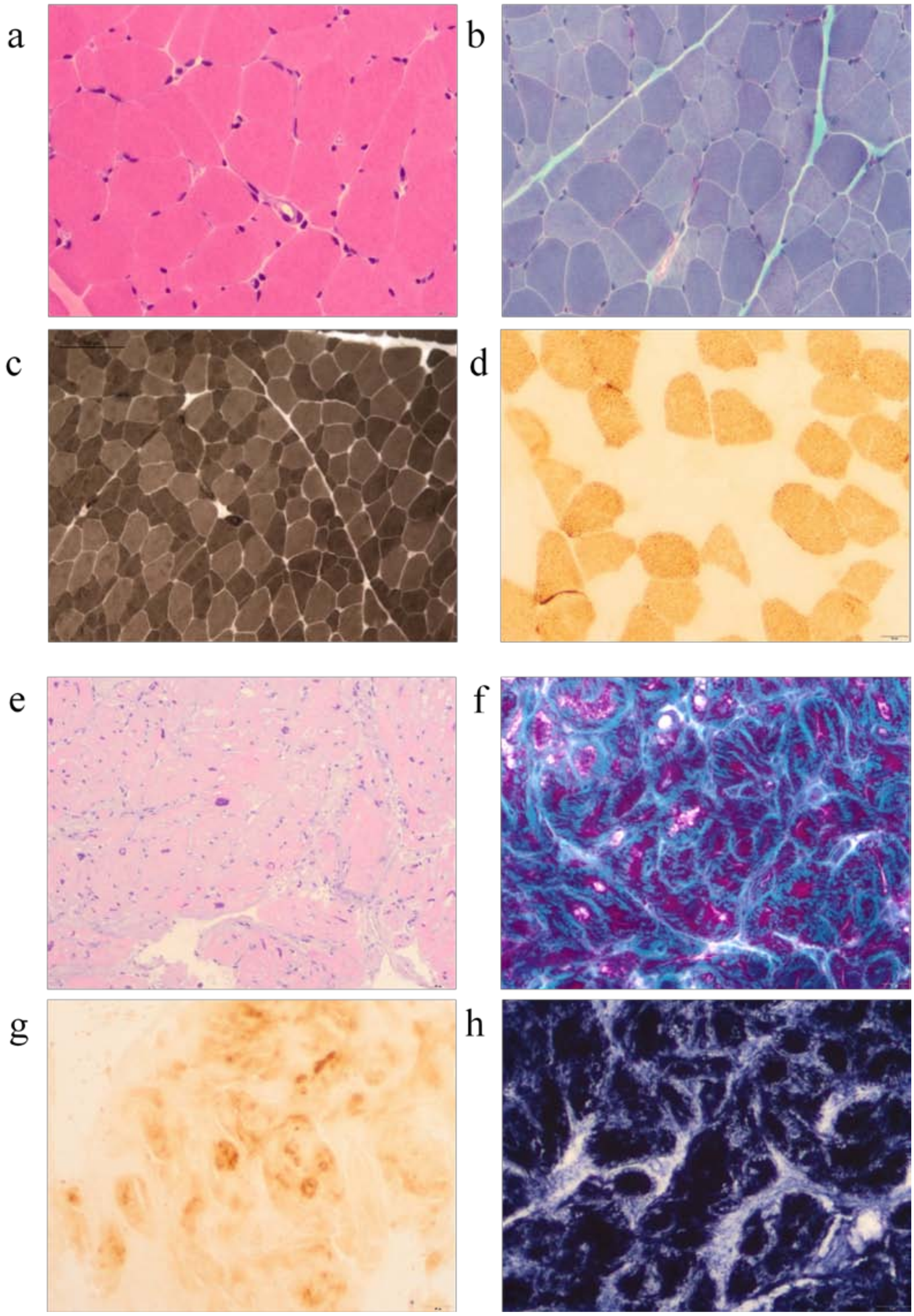

Supplementary Figure 12: Macroscopic analysis of Patient 4 muscle biopsies. (a-d) Quadriceps muscle biopsy. Haematoxylin & Eosin (a), modified Gomori trichrome (b) and ATPase pH 9.4 (c) staining demonstrated expected variability in muscle fibre size, with isolated internal nuclei, and selective fiber type 2 atrophy. COX staining (d) showed weak mosaic COX fibers. (e-h) Myocardium biopsy. Haematoxylin & Eosin (e) demonstrated marked variability in myocardiocytes and prominent myonuclei. Modified Gomori trichrome staining (f) showed presence of “ragged red” fibers, consistent with a mitochondrial citopathy. SDH staining (g) showed mitochondrial proliferation with uniformly prominent SDH-positive fibres. COX staining (h) showed a widespread reduction.

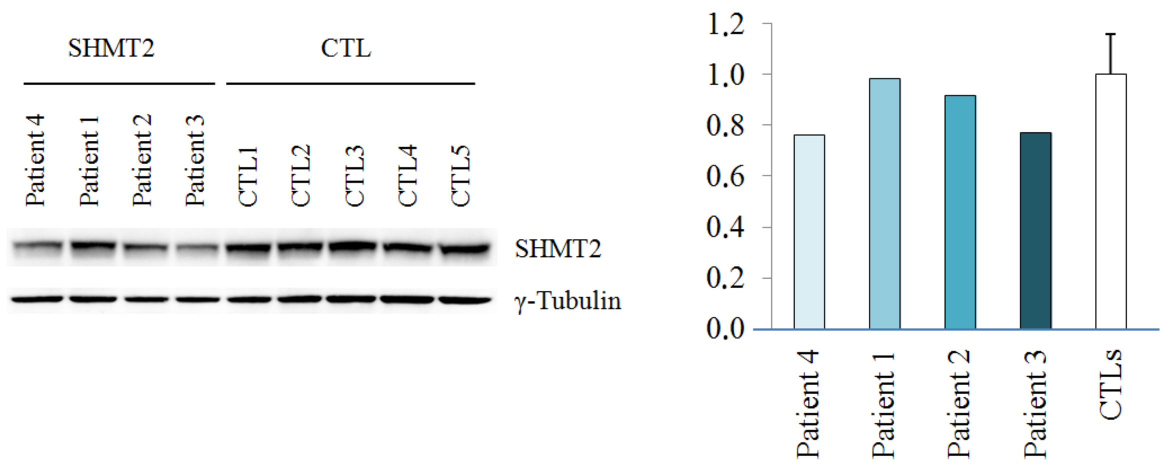

Supplementary Figure 13: Western Blot showing SHMT2 protein levels in fibroblasts from patients (n=4) and controls (n=5). Right, quantification and normalization of SHMT2 levels relative to γ-tubulin levels and to pooled controls. Values of controls are expressed as mean±SD.

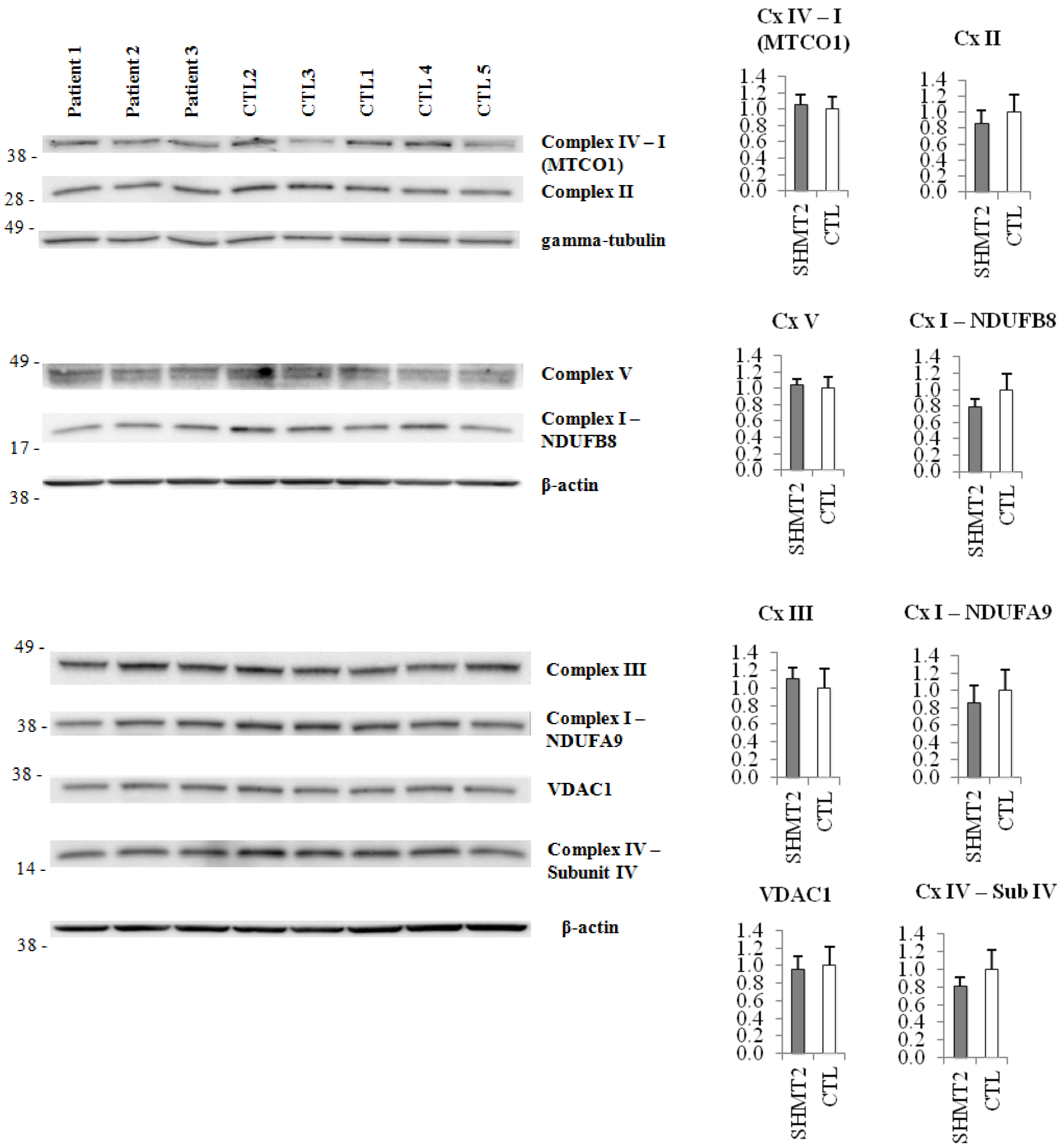

Supplementary Figure 14: Western blots showing protein levels of mitochondrial respiration complexes in fibroblasts from 3 patients and 5 controls. Quantification was performed by normalization to β-actin or γ-tubulin levels. WB was performed using 12% polyacrylamide gels, charging 25 μg protein. Values are expressed as mean±SD.

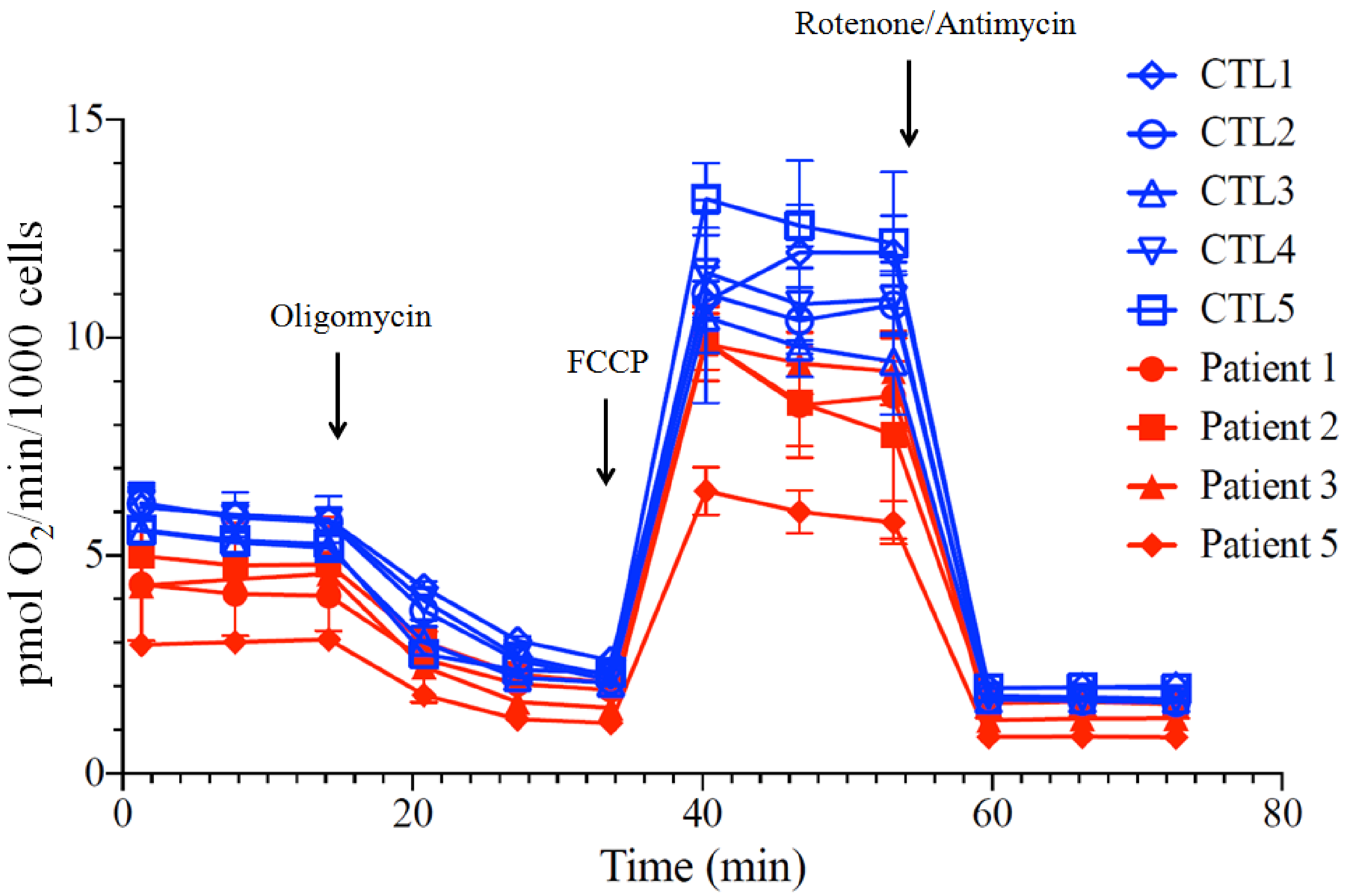

Supplementary Figure 15: Seahorse respirometry traces for control (n=5) and patient (n=4) fibroblasts. Sequential injections of oligomycin (1μM), FCCP (2μM) and rotenone/antimycin (1μM) are indicated.

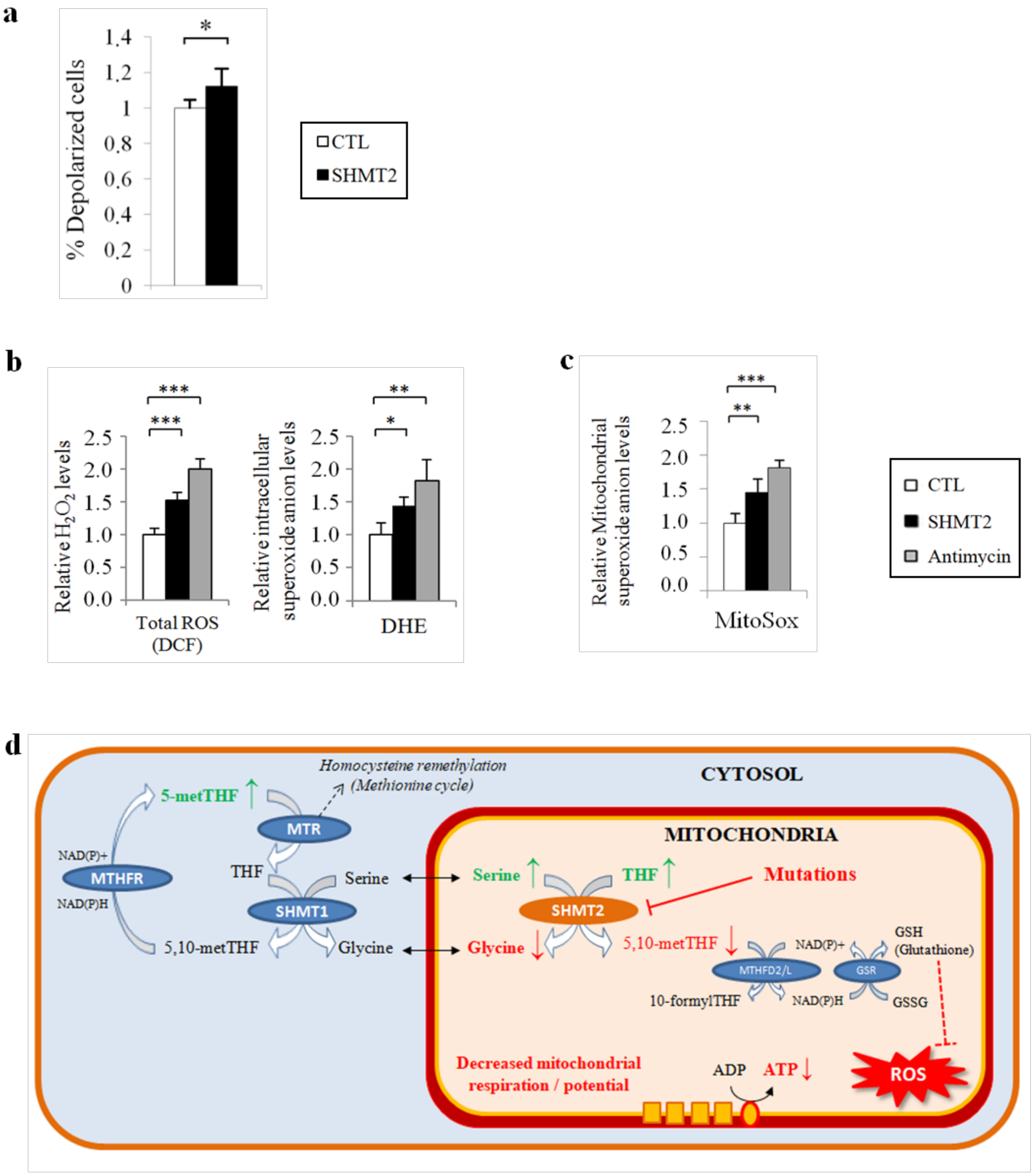

Supplementary Figure 16: Functional characterization of fibroblasts from SHMT2-deficient patients. a) Inner mitochondrial membrane potential quantification. Percentages of depolarized cells were measured in fibroblasts from control individuals (CTL, n=6) and patients (SHMT2, n=5). Values are expressed as the mean±SD (*:p<0.05, unpaired Student's t-test). b) Evaluation of intracellular ROS levels. Intracellular H2O2 (DCF probe) and intracellular superoxide anion (DHE probe) were quantified in fibroblasts from control individuals (CTL, n=6) and patients (SHMT2, n=4). Antimycin (200 µM for 1 h) was used as positive control in all experiments. Quantification depicted as the fold change compared to control fibroblast levels. Values are expressed as mean±SD (*P<0.05, **P<0.01 and ***p<0.001 one-way ANOVA followed by Sidak's post hoc test). c) Quantification of intracellular mitochondrial superoxide anion (MitoSOX probe) in fibroblasts from control individuals (CTL, n=6) and patients (SHMT2, n=5). Antimycin (200 µM for 1 h) was used as positive control. Quantification depicted as the fold change compared to control fibroblast levels. Values are expressed as mean±SD (**:p<0.01, ***:p<0.001, one-way ANOVA followed by Sidak's post hoc test). d) Schematic representation of SHMT2 mutations' consequences.

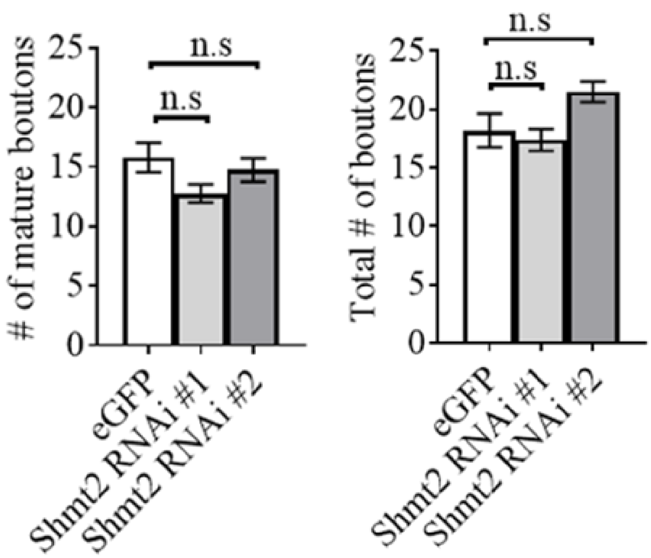

Supplementary Figure 17: Quantification of the average number of mature and total boutons in control (eGFP) and mutant (Shmt2 RNAi #1, #2) flies. n=15-18 for group. Values are expressed as mean±SEM, and one-way ANOVA with Tukey's multiple comparisons test was performed. n.s.: not significant.
